# The *Mycetophila ruficollis* Meigen (Diptera, Mycetophilidae) group in Europe: elucidating species delimitation with COI and ITS2 sequence data

**DOI:** 10.3897/zookeys.508.9814

**Published:** 2015-06-15

**Authors:** Siiri Jürgenstein, Olavi Kurina, Kadri Põldmaa

**Affiliations:** 1 Institute of Agricultural and Environmental Sciences, Estonian University of Life Sciences, Kreutzwaldi st 5D, 51014 Tartu, ESTONIA; 2 Department of Botany, Institute of Ecology and Earth Sciences, University of Tartu, Ravila 14a, 50411 Tartu, ESTONIA

**Keywords:** Fungus gnats, *Mycetophilini*, mycetophages, morphology, phylogenetic analysis, taxonomy, DNA barcoding

## Abstract

European species of the *Mycetophila ruficollis* group are compared on the basis of morphology and sequences of mitochondrial cytochrome oxidase subunit one (COI) and the ITS2 region of nuclear ribosomal DNA. The study represents the first evaluation of morphology-based species delimitation of closely related fungus gnat species by applying molecular information. Detailed descriptions and illustrations of the male terminalia are presented along with a key for the identification of all nine European species of the group. Phylogenetic analyses of molecular data generally supported the morphological species discrimination. The barcoding region of COI superseded ITS2 rDNA in resolving species. In the COI barcoding region interspecific differences ranged from 2.9 to 10.6% and the intraspecific distance from 0.08 to 0.8%. Only COI data distinguished between the similar and closely related *Mycetophila ichneumonea* and *Mycetophila uninotata* of which the latter was observed to include cryptic species. The host range of some species is suggested to be narrower than previously considered and to depend on the forest type. Presented evidence indicates the importance of analysing sequence data of morphologically very similar mycetophages reared from identified host fungi for elucidating species delimitation as well as their geographic and host ranges. New country records, viz. Estonia for *Mycetophila evanida*, Georgia for *Mycetophila ichneumonea*, *Mycetophila idonea* and *Mycetophila ruficollis*, and Norway for *Mycetophila strobli*, widen the known distribution ranges of these species.

## Introduction

*Mycetophila* Meigen, 1803 is one of the largest and earlier described genera among fungus gnats (Diptera: Mycetophilidae). The first fungus gnat ever described is today known as *Mycetophila fungorum* (De Geer, 1776), a widespread and common species in the Palaearctic region. Since then more than 650 species from all biogeographical realms have been described in the genus (Bechev 2000, Zaitzev 2003, Oliveira and Amorim 2014). Based on morphological characters, the genus has been divided into several subgenera (particularly in the Neotropical region: cf. Lane 1955) and species-groups (particularly in the Holarctic region: cf. Laffoon 1957, Laštovka 1963, 1972, Zaitzev 1999). However, neither analyses addressing the intrageneric phylogeny nor keys to all species have yet been provided, with the most exhaustive presented by Laffoon (1957) for the Nearctic species and Zaitzev (2003) for the Palaearctic species.

**Figures 1–3.**
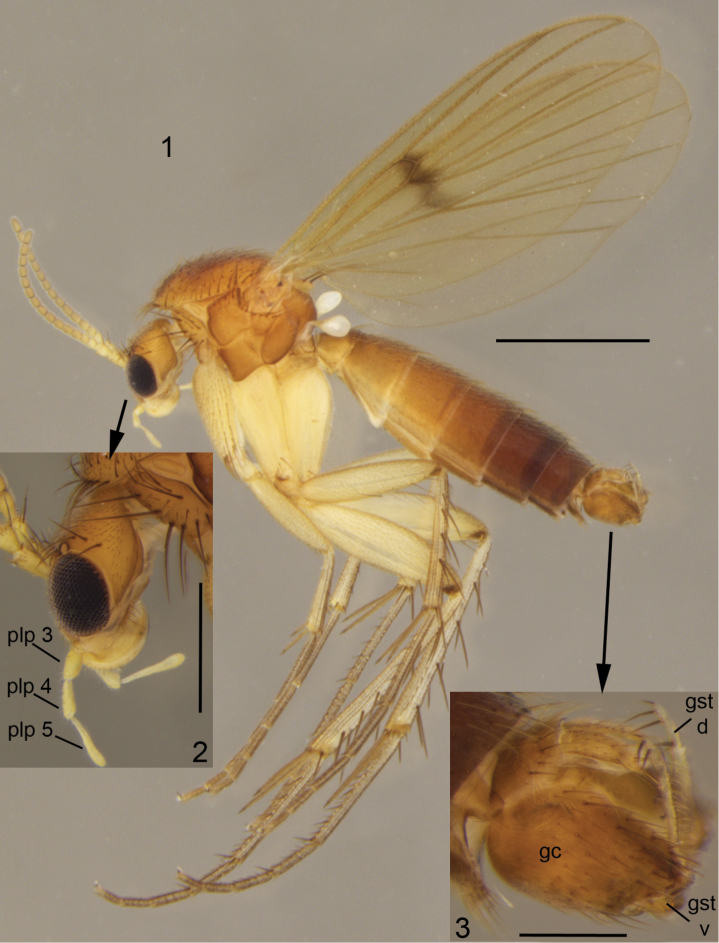
*Mycetophila ichneumonea* Say, 1823, a typical member of the *Mycetophila ruficollis* group. **1** male habitus **2** head with maxillary palpi, closer view **3** male terminalia, closer view. Scale bar = 1 mm (**1**), 0.5 mm (**2**) and 0.2 mm (**3**). Abbreviations: plp = segments of maxillary palpus; gc = gonocoxite; gst d = dorsal branch of gonostylus; gst v = ventral branch of gonostylus.

One of the most clearly delimited and supposedly monophyletic intrageneric subdivisions is the *Mycetophila ruficollis* Meigen species-group, introduced by Laštovka (1972). Members of the group (see Figs 1–3) are morphologically characterised by 1) mid tibiae without ventral bristles, 2) bM-Cu setose, with 8 or more setae and 3) wings with central spot only (except one Nearctic species; Laštovka and Kidd 1975). The general outline of male terminalia and absence of ventral setae on mid tibia are shared with *Mycetophila fungorum* and allied species, which are otherwise devoid of setae on bM-Cu and form another intrageneric group of species. Within the limits of the *ruficollis*-group, there are 19 currently recognised species: 17 of them are from the Holarctic (Laštovka 1972, Laštovka and Kidd 1975, Chandler and Ribeiro 1995, Wu 1997) and two from the Oriental region (Wu 1997). The records of *Mycetophila ruficollis* Meigen, 1818 in the Afrotropical region are based probably on misidentifications (cf. Matile 1980) and represent obviously undescribed species. Laštovka (1972) discussed about 30 closely related species in the group which also include supposedly undescribed species, especially those from the Oriental region, known to him at that time. Eleven Holarctic species were covered by a detailed study by Laštovka (1972) including a key to species, while seven species from the Palaearctic region were described subsequently by Laštovka and Kidd (1975), Chandler and Ribeiro (1995) and Wu (1997). All species with known biology are mycetophagous in their larval stage, colonising a variety of Agaricales, Russulales and to lesser extent Boletales, with one species reared from Polyporales (e.g. Yakovlev 1994, Ševčík 2010). However, little is known about the host range of species in this group. On the basis of published data, their larvae seem to be most frequent in fruit bodies of *Russula*, *Lactarius*, *Cortinarius* and *Pholiota*, tending to avoid species of Boletales (e.g. Hackman and Meinander 1979, Krivosheina et al. 1986, Kurina 1994, Ševčík 2010, Põldmaa et al. 2015).

The *Mycetophila ruficollis* species-group includes morphologically similar species which are reliably identifiable only by comparing details of male terminalia. While discussing intraspecific variability, Laštovka (1972) noted that in spite of variable coloration of the body, the characters in male terminalia are generally constant but vary in some details. Moreover, Laštovka and Kidd (1975) described *Mycetophila britannica* in two morphological forms and noted that *Mycetophila ichneumonea* may be polytypic consisting of 2–3 subspecies. This led us to suggest that the species-group may hide some undiscovered diversity, i.e. cryptic species with possibly different larval diet.

One of the most important and frequently used set of characters for delimiting cryptic species is that obtained from DNA sequence analyses. In studies of fungus gnats, DNA sequence data are so far mostly used to clarify phylogenetic relationships of subfamilies and/or genera (e.g. Rindal et al. 2009, Martinsson et al. 2011, Ševčík et al. 2014) but also to associate sexes of one species (Kurina et al. 2011) and compensate for the deficiencies of the morphological component in species identification of otherwise obscure material (Põldmaa et al. 2015). For delimiting species, molecular characters have thus far been incorporated only in the genus *Neuratelia* (Kurina et al. 2015). In addition to the widely used ’DNA barcode’ fragment (COI) (e.g. Hebert et al. 2003, Hajibabaei et al. 2006), ITS2 sequences have successfully been applied for species delimitation. The aim of this study was to 1) test morphological species delimitation by the application of molecular methods, 2) present a key to European species of the *Mycetophila ruficollis* group supplemented by modern illustrations of male terminalia, and 3) search for possible cryptic species. Because this group includes morphologically extremely similar species, they are frequently being identified only to species-group level even by specialists. Our intention was to provide also a reliable DNA reference dataset that could be used in further DNA based identification of fungus gnats.

## Material and methods

### Morphological analyses

The study is based on material collected throughout the Europe during 1984 to 2014 mostly by Malaise traps, light traps and sweepnetting. In addition, several specimens from Lebanon and Georgia are also included. A part of the Estonian material was reared from macrofungi. For that, fruit bodies were isolated into plastic containers and covered with nylon gauze, while peat was used as a pupation substrate. Containers were incubated in a lab facility and checked every other day while emerged adults were collected by an aspirator (see also Põldmaa et al. 2015). Altogether 116 male specimens of the *Mycetophila ruficollis* group were morphologically studied.

The majority of the included material was initially collected into 70 % ethyl alcohol and studied under stereomicroscopes Olympus SZ61or Leica S8APO. For detailed study of male terminalia they were detached and macerated in a solution of KOH followed by neutralization in acetic acid and washing in distilled water (for details see Kurina 2003). The remaining chitinous structure was thereafter separated to several anatomical units which were: 1) inserted into glycerine for study and preserved as glycerine preparations or 2) slide-mounted individually in Euparal between two pieces of coverslip allowing them to be studied from both sides under a compound microscope (for details see Hippa and Kurina 2012). The preservation method of each specimen is indicated in the material sections. The habitus photo was taken from a specimen in alcohol. All photos of male terminalia were taken from preparations in Euparal and combined by software LAS V.4.1.0. from multiple gradually focused images taken by a camera Leica DFC 450 attached to the compound microscope Leica DM 6000 B (see also Kurina and Oliveira 2013). Morphological terminology follows generally that of Søli et al. (2000) while several specific terms of male terminalia (see Table 1) are used according to Laštovka (1972). The term “bristle” is used for a seta that is significantly larger in length and diameter than surrounding setae (see also Merz and Haenni 2000). Detailed revised descriptions of male terminalia figured by Laštovka (1972) and Laštovka and Kidd (1975), supplemented by illustrations, are provided herein.

**Table 1.** Terminology used for describing male terminalia with synonyms from earlier studies and references to corresponding figures.

Present study	Laštovka 1972, Laštovka and Kidd 1975	Corresponding figures and used abbreviations
gonocoxite	gonocoxopodite	Figs 3, 33, 34 – gc
posterior margin of gonocoxite	posterior margin of gonocoxopodite	Fig. 34 – gc pm
posterior impression of gonocoxite	posterior impression of gonocoxopodite	Fig. 34 – gc pi
anterior impression of gonocoxite	anterior impression of gonocoxopodite	Fig. 34 – gc ai
dorsal branch of gonostylus	dististyle	Fig. 3 – gst d; Figs 6–14
ventral branch of gonostylus	basistyle	Fig. 3 – gst v; Figs 15–32
posterior margin, lateral margin, basal margin and basal angle of dorsal branch of gonostylus	posterior margin, lateral margin, basal margin and basal angle of dististyle	Fig. 6 – pm, lm, bm, ba
distal posterior process and proximal posterior process of dorsal branch of gonostylus	distal posterior process and proximal posterior process of dististyle	Fig. 6 – dpp, ppp
medial bristle of dorsal branch of gonostylus		Fig. 6 – mb
posterior process of ventral branch of gonostylus	posterior process of basistyle	Figs 15, 16 – pp
spines 1–4 on the ventral branch of gonostylus	spines 1–4 on the basistyle	Figs 15, 16 – sp 1, sp 2, sp 3, sp 4
aedeagal complex	intromittent organ	Figs 33–44
aedeagus	aedeagus	Figs 33, 35 – aed
ejaculatory apodeme	penis tube	Fig. 35 – ej ap, ej ap b
rim of ejaculatory apodeme	rim of penis tube	Fig. 35 – ej ap r
aedeagal guide	penis sheath	Fig. 33 – aed gd
lateral impression on aedeagal guide	lateral impression on penis sheath	Fig. 33 – aed gd li
aedeagal apodeme	thecal apodeme	Figs 33, 35 – aed ap

The following acronyms are used for depositories:

IZBE Institute of Agricultural and Environmental Sciences, Estonian University of Life Sciences [former Institute of Zoology and Botany], Tartu, Estonia;

JSPC Jukka Salmela private collection (Rovaniemi, Finland);

PCPC Peter Chandler private collection (Melksham, United Kingdom).

### Molecular techniques

Based on the preliminary morphological determination, at least one male specimen from every species from each locality was allocated for DNA sequencing. For that, after detaching terminalia, the rest of the abdomen or a leg was placed in the lysis buffer, preserving the rest of the specimen. DNA was extracted by incubating the material overnight at 56 °C in 10X Reaction Buffer B (Solis Biodyne, Tartu, Estonia) with the addition of 2.5 μl (20 mg/ml) proteinase K (Fermentas, Lithuania). After 15 min at 98 °C the material was centrifuged and DNA solution pipetted into a new tube.

In 66 specimens, the 658 bp barcode region at the 5’ end of the mitochondrial cytochrome oxidase subunit I (COI) gene was amplified and sequenced with primers Lep-F1 and Lep-R1 (Hebert et al. 2004) or LCO1490 and HCO2198 (Folmer et al. 1994). In most of the specimens an additional 790 bp, following the barcoding region, were amplified and sequenced with primers C1-J-2195 and TL2-N-3014 (Simon et al. 1994). In 33 specimens, the second fragment of the internal transcribed spacer region (ITS2) was amplified and sequenced using primers ITS2A and ITS2B (Beebe and Saul 1995). PCR was performed in a total volume of 25μl, with the reaction mixture containing 1X HOT FIREPol®Blend Master Mix Ready to Load (Solis BioDyne, Tartu, Estonia), 10 pmol of primers and 20–80 ng of DNA. PCR was carried out in an Eppendorf Mastercycler epigradient thermocycler (Eppendorf AG, Hamburg, Germany). The initial denaturation at 95 °C for 15 min was followed by 35 cycles of 30 s at 95 °C, 30 s at 45–60 °C (depending on primers) and 1 min at 72 °C, followed by a final extension at 72 °C for 10 min. PCR products were visualised on a 1.2% agarose gel, and the remaining PCR product was purified with fast alkaline phosphatase and exonuclease I (Thermo Scientific, Pittsburgh, USA). DNA sequencing was performed at Macrogen Europe (Amsterdam, Netherlands) or at the Estonian Biocentre (Tartu, Estonia). All sequences obtained in this study were deposited in GenBank under accession numbers KR997602–KR997703.

### Phylogenetic analyses

The sequences were edited and assembled with Sequencher 5.1 (Gene Codes, Ann Arbor, MI, USA), aligned with Mafft 6 online version (Katoh and Toh 2008) and edited manually using GeneDoc 2.6.0.3. Phylogenetic analyses were performed on the separate and combined COI and ITS2 datasets. Four sequences of *Mycetophila fungorum* were used as an outgroup in all analyses. Bayesian analyses were performed on all three datasets using MrBayes at the Cipres website (http://www.phylo.org) with default settings of the online version and invgamma model. Each analysis was run for 10 mln generations, of which every 1000th was sampled. The first 25% of sampled trees were discarded as burn-in. Posterior probabilities were calculated from remaining 7500 trees. Parsimony analyses were performed in PAUP (Swofford 2003). During the 1000 random searches 50 best trees were kept in each search that were all used for additional swapping. This was done until the limits of available computer memory was reached. The most parsimonious trees obtained were used to calculate the strict consensus. 1000 bootstrap replications were performed to assess branch support. Intra- and interspecific distance variance was calculated in MEGA6 (Tamura et al. 2013) using Kimura 2-parameter model (see e.g. Waugh 2007, Õunap and Viidalepp 2009).

## Results

Based on the morphology, mainly that of male terminalia, the studied material was identified to belong to all nine species of the *Mycetophila ruficollis* group known from Europe. Deviation in morphological characters of some specimens suggested that these might represent additional undescribed species. Phylogenetic analyses, based on molecular data, recognised seven well supported clades, corresponding to the morphologically distinguished species. Fresh material, suitable for molecular analyses, was not available for two European species: *Mycetophila sepulta* and *Mycetophila suffusala*. The phylogenies led to reconsideration of morphology-based identification in several specimens. Consequently, the colouration and dimensions of the gnats’ body were realised to be variable but the characters of male terminalia mostly constant within a species.

The interspecific genetic distance among species of the *Mycetophila ruficollis* group was calculated for the COI barcoding region, including 647 bp (Table 2). Interspecific differences ranged from 2.9% (between *Mycetophila ichneumonea* and *Mycetophila uninotata*) to 10.6% (between *Mycetophila evanida* and *Mycetophila idonea*), with the mean interspecific distance of 8.1%. The intraspecific distance ranged from 0.08 to 0.8% except for 2.3% in *Mycetophila uninotata*. The second region of COI, following the barcoding region, distinguished the seven analysed species based on 4.0–9.3% of interspecific variation (data not shown).

**Table 2.** Genetic distances between species of the *Mycetophila ruficollis* group and *Mycetophila fungorum*, used as an outgroup, quantified according to the Kimura 2-parameter model from the COI barcoding region.

		1	2	3	4	5	6	7	8
1	*Mycetophila ichneumonea*	**X**							
2	*Mycetophila uninotata*	2.9%	**X**						
3	*Mycetophila strobli*	8.2%	8.1%	**X**					
4	*Mycetophila ruficollis*	8.8%	9.3%	7.2%	**X**				
5	*Mycetophila evanida*	7.3%	7.2%	6.7%	7.4%	**X**			
6	*Mycetophila britannica*	8%	7.8%	6.9%	8.1%	6.6%	**X**		
7	*Mycetophila idonea*	9.9%	9.5%	9.8%	9.7%	10.6%	10.1%	**X**	
8	*Mycetophila fungorum*	11.1%	11.7%	11.8%	12.3%	11.9%	12.5%	12.4%	**X**

The COI datamatrix comprised 66 sequences and 1432 characters (1108 constant, 287 parsimony informative). The ITS2 datamatrix comprised 37 sequences and 584 characters of which 535 were constant and 47 parsimony informative. The combined COI and ITS2 datamatrix comprised 37 sequences and 2016 characters (1662 constant, 331 parsimony informative). When comparing the ITS2 and COI regions in the 37 sequences for which both data were available, the proportion of variable sites in COI (21.3%) exceeded that in ITS2 (8.4%) more than twice. In the COI dataset of 66 sequences variable sites represented 22.6% of the total amount, with the first (barcoding) part of 647 basepairs (available for 61 sequences) including 29.9% and the following 785 basepairs (47 sequences) 22.3% of variable sites.

**Figure 4.**
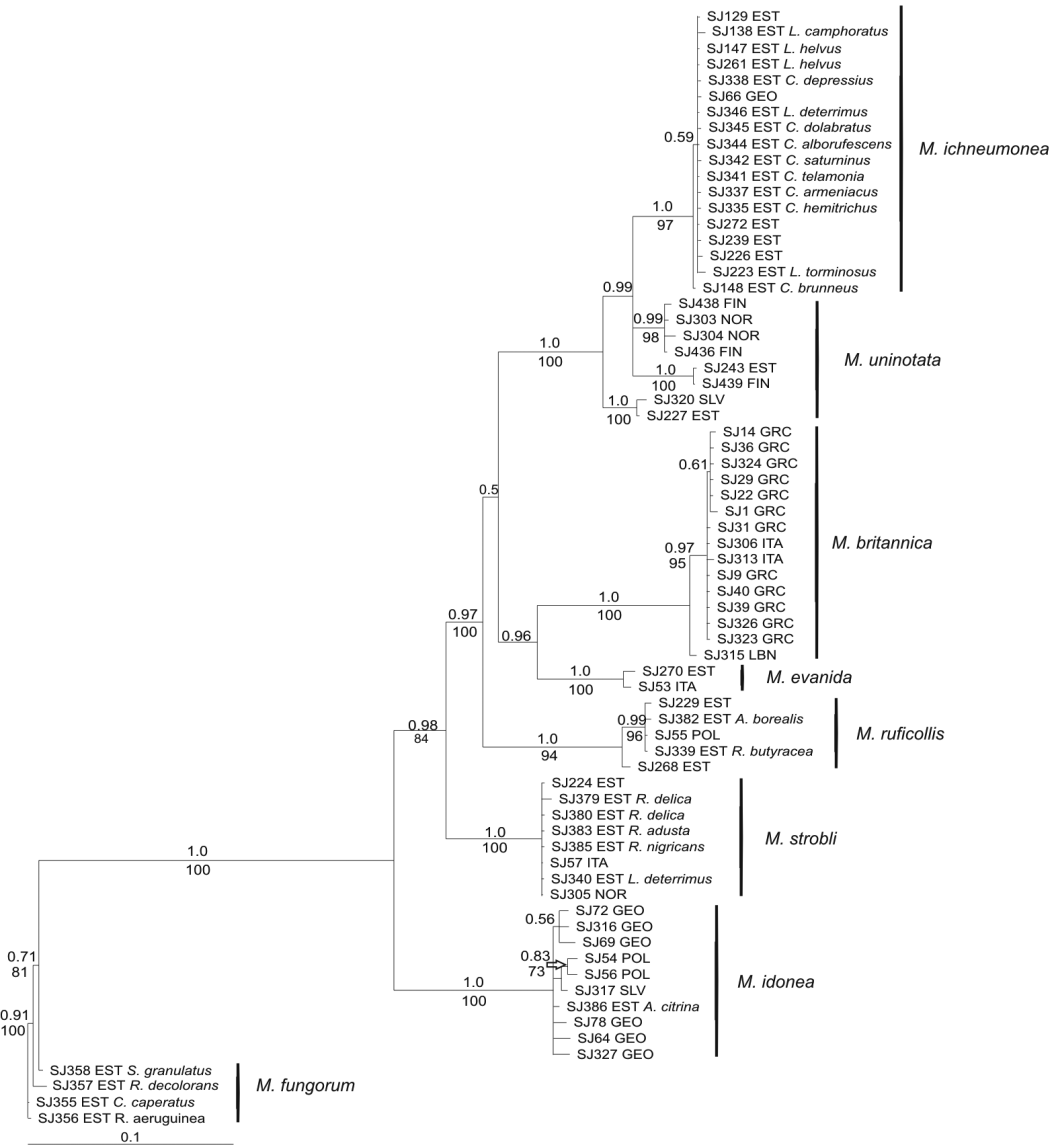
Bayesian consensus tree of the COI regions of *Mycetophila ruficollis* species group. Posterior probability values are presented above the branches and bootstrap support values below the branches. Scale bar indicates substitutions per site. For gnats reared from fungal fruitbodies, the host is indicated.

**Figure 5.**
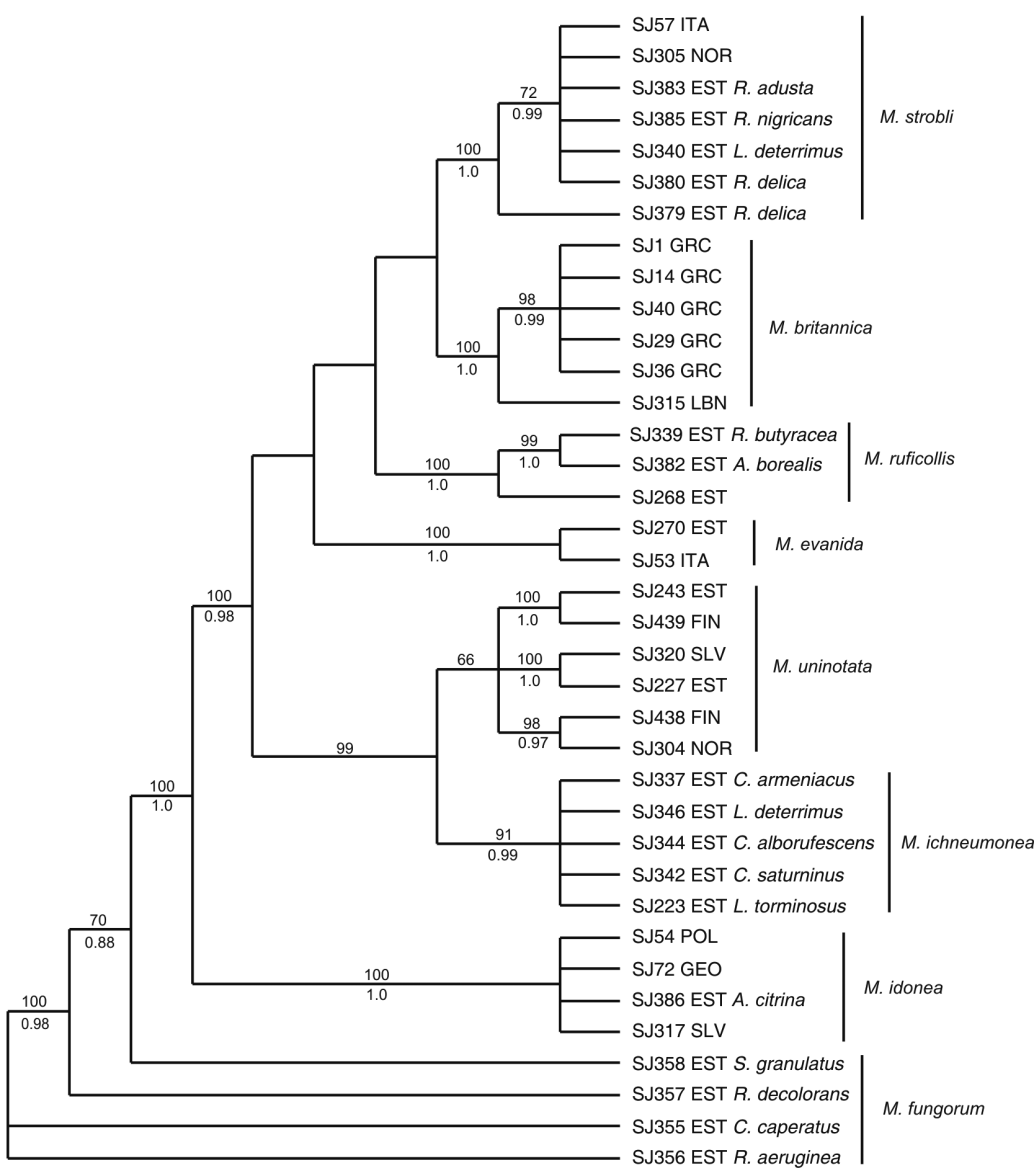
Consensus of most parsimonious trees calculated from combined COI and ITS2 rDNA sequence data of the *Mycetophila ruficollis* species group. Bootstrap support values are presented above the branches and posterior probability values below the branches. For gnats reared from fungal fruitbodies, the host is indicated.

Phylogenetic reconstructions of COI data (Fig. 4) distinguished six strongly supported species (bootstrap support 94% to 100%, posterior probabilities 0.94-1.0) in the ingroup. However, *Mycetophila uninotata* appeared paraphyletic with three strongly supported lineages. The analyses of COI data also support close relationship between *Mycetophila ichneumonea* and *Mycetophila uninotata* as well as *Mycetophila evanida* and *Mycetophila britannica*. By contrast, the ITS2 trees (not shown) were much less resolved, with four well-supported groups recognised. The ITS2 phylogenies did not distinguish *Mycetophila ichneumonea* from *Mycetophila uninotata* with one *Mycetophila strobli* specimen from Italy also placed in this clade. The consensus of most parsimonious (Fig. 5) as well as the Bayesian trees calculated from 37 combined COI and ITS2 sequences were well resolved and with higher support values for most of the clades than obtained in analyses of individual gene regions. The main difference was observed in the three lineages of *Mycetophila uninotata* forming a monophyletic group, yet receiving only low support.

Members of the *Mycetophila ruficollis* species group were selected from the material of adults reared from mushrooms collected from Estonia during 1988-1990 (Kurina 1991) and extensive sampling in five pine dominated boreal forests in 2011 (Põldmaa et al. 2015) as well as scattered localities in the following years. Among the >11 000 adults reared from the pine forest material most gnats from the *Mycetophila ruficollis* species group were sequenced and identified as belonging to *Mycetophila ichneumonea*. Among the 100 fungal species, this gnat emerged only from 12 fruit bodies of *Lactarius* and 4 fruit bodies of *Cortinarius*. Only one specimen, reared from *Rhodocollybia butyracea*, represented *Mycetophila ruficollis*. The study presents new species records for Estonia (*Mycetophila evanida*), Georgia (*Mycetophila ichneumonea*, *Mycetophila idonea*, *Mycetophila ruficollis*) and Norway (*Mycetophila strobli*).

### Key to the species of the *Mycetophila ruficollis* group in Europe based on characters of male terminalia

The key is compiled on the basis of original data, Laštovka (1972), and Laštovka and Kidd (1975).

**Table:** 

1	4^th^ palpal segment wider than the 3^rd^ and about twice as wide as 5^th^, and about as long as the 5^th^ (Laštovka and Kidd 1975: Fig. 1). Posterior margin of gonocoxite ventromedially undulating or with diminutive central prominence	**2**
–	4^th^ palpal segment about as wide as the 3^rd^ and only slightly wider than the 5^th^, and distinctly shorter than the 5^th^ (Laštovka and Kidd 1975: Figs 7–15). Posterior margin of gonocoxite ventromedially convex or with a clear prominence	**3**
2	Wing with central spot only. Posterior margin of gonocoxite ventromedially undulating (Fig. 42). Lateral margin of dorsal branch of gonostylus almost straight (Fig. 10)	***Mycetophila ruficollis* Meigen**
–	Wing with central spot and apical dark shade. Posterior margin of gonocoxite ventromedially with diminutive central prominence which is somewhat sunken into the posterior impression (Figs 47, 48). Lateral margin of dorsal branch of gonostylus with clear concavity (Fig. 13)	***Mycetophila suffusala* Chandler & Ribeiro**
3	Central spot of wing narrow and indistinct (sometimes almost absent). Dorsal branch of gonostylus with: lateral margin almost stright and distal posterior process subequal to proximal posterior process, both separated by wide and deep notch (Fig. 9)	***Mycetophila sepulta* (Laffoon)**
–	Central spot of wing distinct. Dorsal branch of gonostylus with different combination of characters: with clear concavity at lateral margin and/or with distal and proximal posterior processes in different height	**4**
4	Posterior impression of gonocoxite wide and compressed with oblique lateral projections (Figs 49, 50); posterior process of ventral branch of gonostylus with well distinguished warts (Fig. 31); posterior processes of dorsal branch of gonostylus about the same height (Fig. 14)	***Mycetophila uninotata* Zetterstedt**
–	Posterior impression of gonocoxite cup-shaped, with vertical lateral projections; posterior process of ventral branch of gonostylus without or with only small warts; posterior processes of dorsal branch of gonostylus unequal in height	**5**
5	Lateral margin of dorsal branch of gonostylus with distinct and deep concavity (Fig. 9). Aedeagal guides distally divided, lateral impressions small or absent (Fig. 37)	***Mycetophila ichneumonea* Say**
–	Lateral margin of dorsal branch of gonostylus without or with a shallow concavity. Aedeagal guides distally only shallowly bifurcated, lateral impressions wide	**6**
6	Posterior margin of dorsal branch of gonostylus sinuate (Fig. 6). Posterior margin of gonocoxite ventromedially concave or with indistinct convexity (Fig. 34)	***Mycetophila britannica* Laštovka & Kidd**
–	Posterior margin of dorsal branch of gonostylus straight. Convexity on posterior margin of gonocoxite ventromedially well outlined	**7**
7	Distance between spines 2, 3 and 4 on ventral branch of gonostylus nearly equal (Figs 27, 18). Posterior process of ventral branch of gonostylus with very small warts. Lateral margin of dorsal branch of gonostylus straight or slightly convex (Fig. 7)	***Mycetophila idonea* Laštovka**
–	Distance between spines 3 and 4 on ventral branch of gonostylus much shorter than distance between spines 3 and 2. Posterior process of ventral branch of gonostylus with warts and setulae. Lateral margin of dorsal branch of gonostylus concave	**8**
8	Posterior margin of gonocoxite laterally from ventromedial convexity slanting (Fig. 46). Posterior margin of dorsal branch of gonostylus proximally from medial bristle with 4 gradually diminishing rather weak bristles (Fig. 12)	***Mycetophila strobli* Laštovka**
–	Posterior margin of gonocoxite laterally from ventromedial convexity straight (Fig. 40). Posterior margin of dorsal branch of gonostylus proximally from medial bristle with 5–6 gradually diminishing strong bristles (Fig. 8)	***Mycetophila evanida* Laštovka**

**Figures 6–14.**
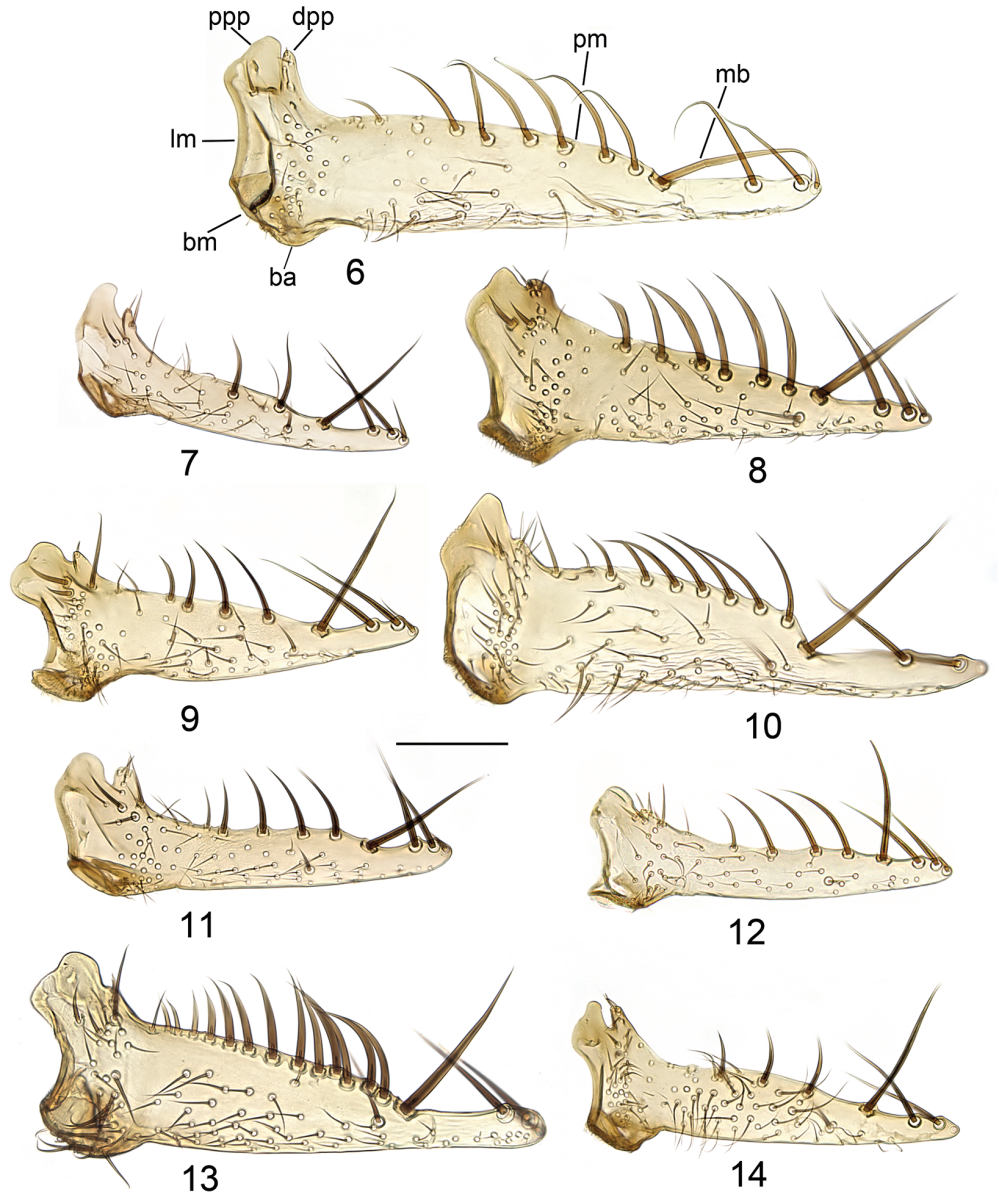
Dorsal branch of gonostylus. **6** *Mycetophila britannica* **7** *Mycetophila idonea* **8** *Mycetophila evanida* **9** *Mycetophila ichneumonea* **10** *Mycetophila ruficollis* **11** *Mycetophila sepulta* **12** *Mycetophila strobli* **13** *Mycetophila suffusala* **14** *Mycetophila uninotata*. Scale bar = 0.1 mm. Abbreviations: ba = basal angle; bm = basal margin; lm = lateral margin; pm = posterior margin; mb = medial bristle; dpp= distal posterior process; ppp = proximal posterior process.

**Figures 15–20.**
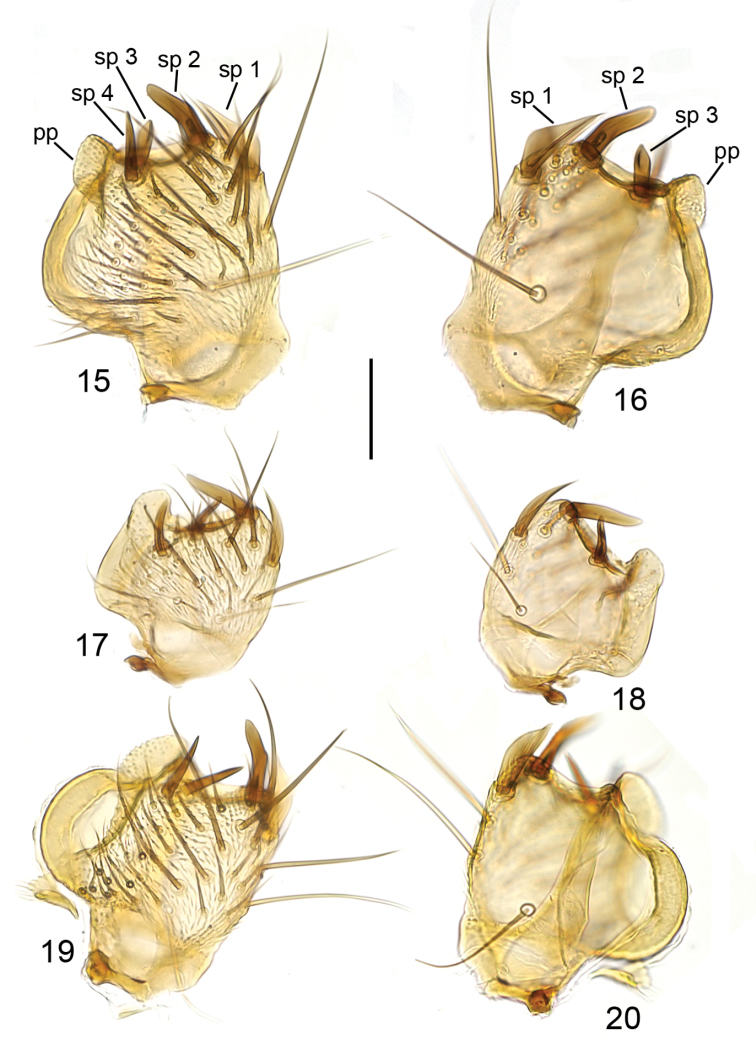
Ventral branch of gonostylus, ventral view (**15, 17, 19**) and internal view (**16, 18, 20**). **15, 16** *Mycetophila britannica* **17, 18** *Mycetophila idonea* **19, 20** *Mycetophila ichneumonea*. Scale bar = 0.05 mm. Abbreviations: pp = posterior process; sp = posterior spines on the ventral branch of gonostylus.

**Figures 21–26.**
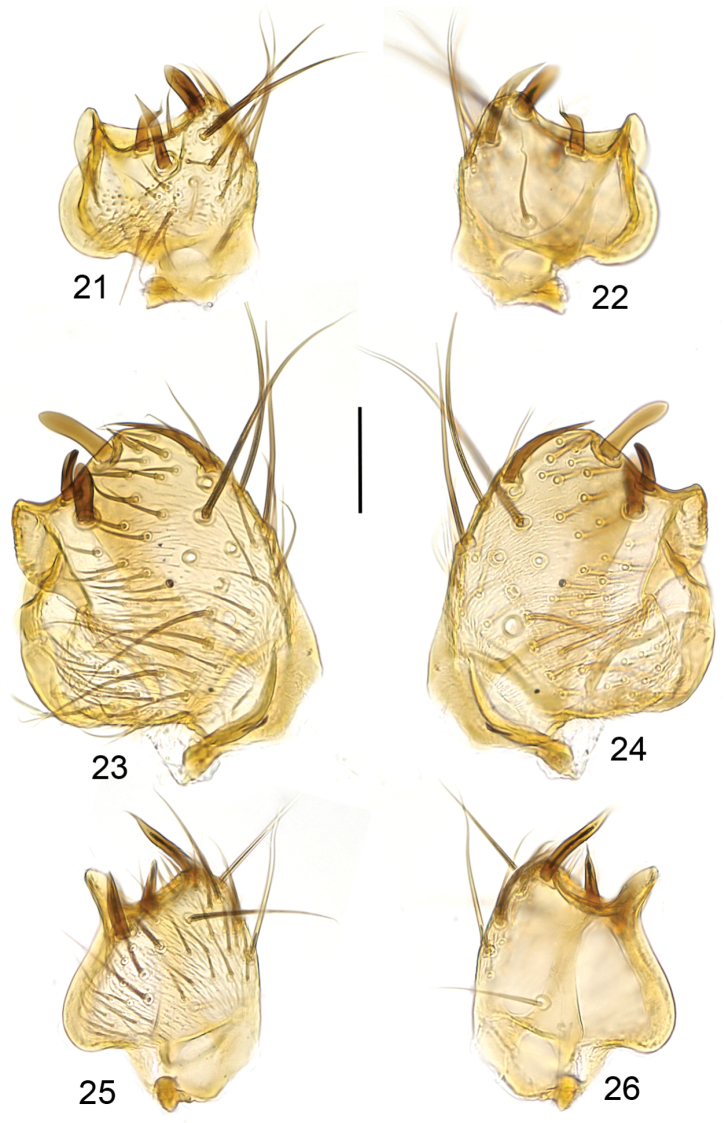
Ventral branch of gonostylus, ventral view (**21, 23, 25**) and internal view (**22, 24, 26**). **21, 22** *Mycetophila evanida* **23, 24** *Mycetophila ruficollis* **25, 26** *Mycetophila sepulta*. Scale bar = 0.05 mm.

**Figures 27–32.**
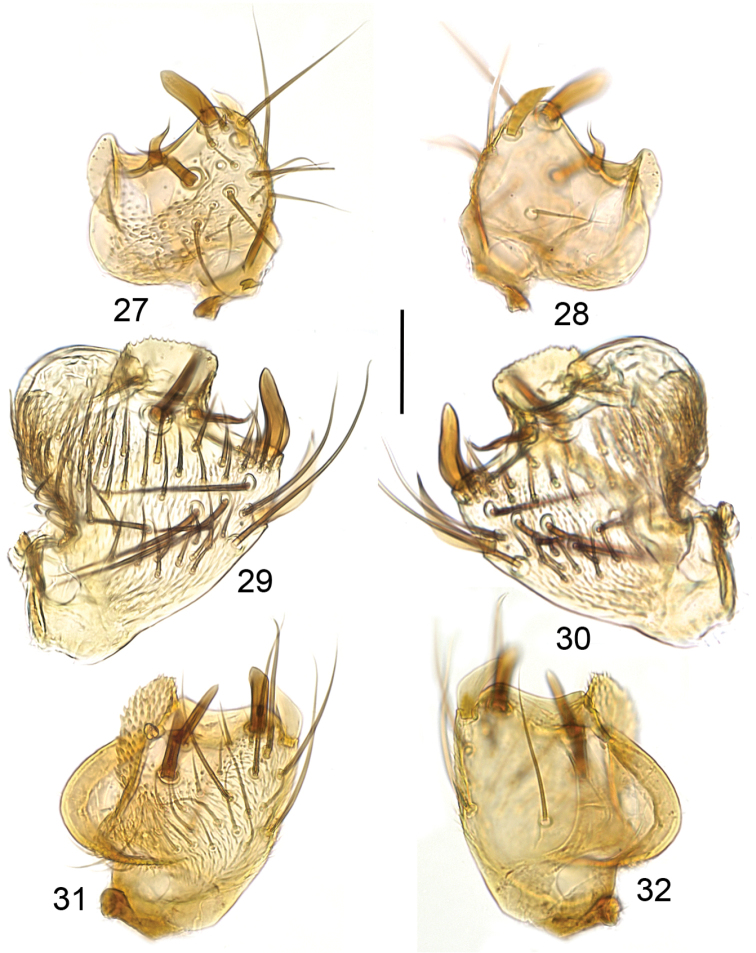
Ventral branch of gonostylus, ventral view (**27, 29, 31**) and internal view (**28, 30, 32**). **27, 28** *Mycetophila strobli* **29, 30** *Mycetophila suffusala* **31, 32** *Mycetophila uninotata*. Scale bar = 0.05 mm.

**Figures 33–38.**
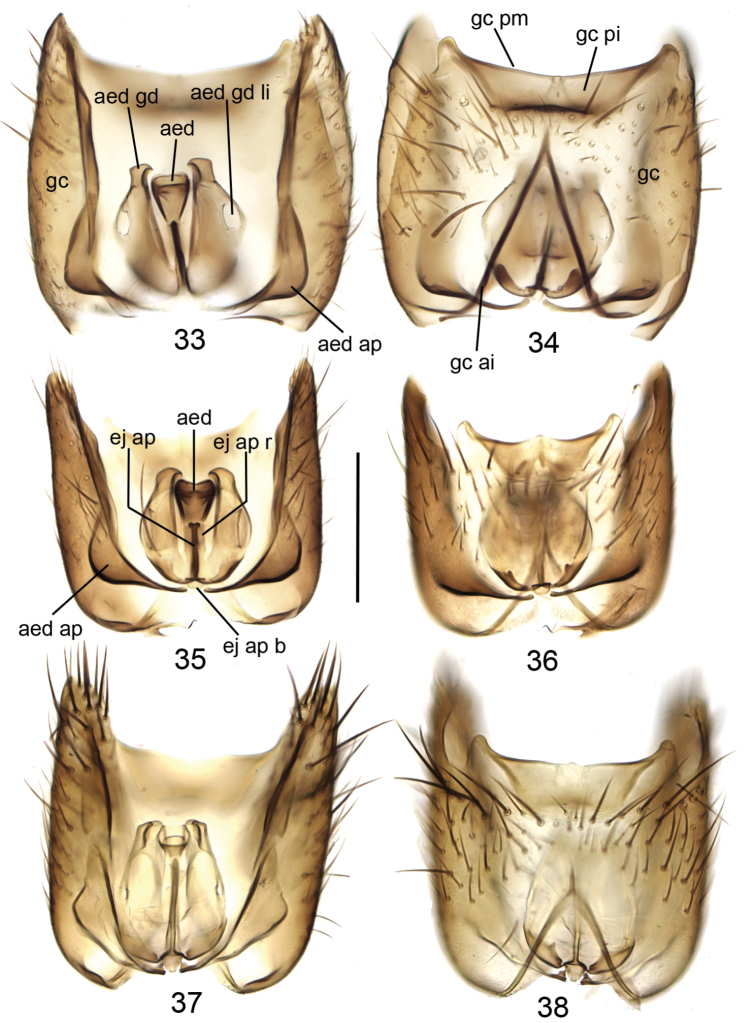
Gonocoxites with aedeagal complex, dorsal view (**33, 35, 37**) and ventral view (**34, 36, 38**). **33, 34** *Mycetophila britannica* **35, 36** *Mycetophila idonea* **37, 38** *Mycetophila ichneumonea*. Scale bar = 0.2 mm. Abbreviations: aed = aedeagus; aed ap = aedeagal apodeme; aed gd = aedeagal guide; aed gd li = lateral impression on the aedeagal guide; ej ap = ejaculatory apodeme; ej ap b = base of ejaculatory apodeme; ej tb r = rim of ejaculatory apodeme; gc = gonocoxite; gc ai = anterior impression of gonocoxite; gc pi = posterior impression of gonocoxite; gc pm = posterior margin of gonocoxite.

**Figures 39–44.**
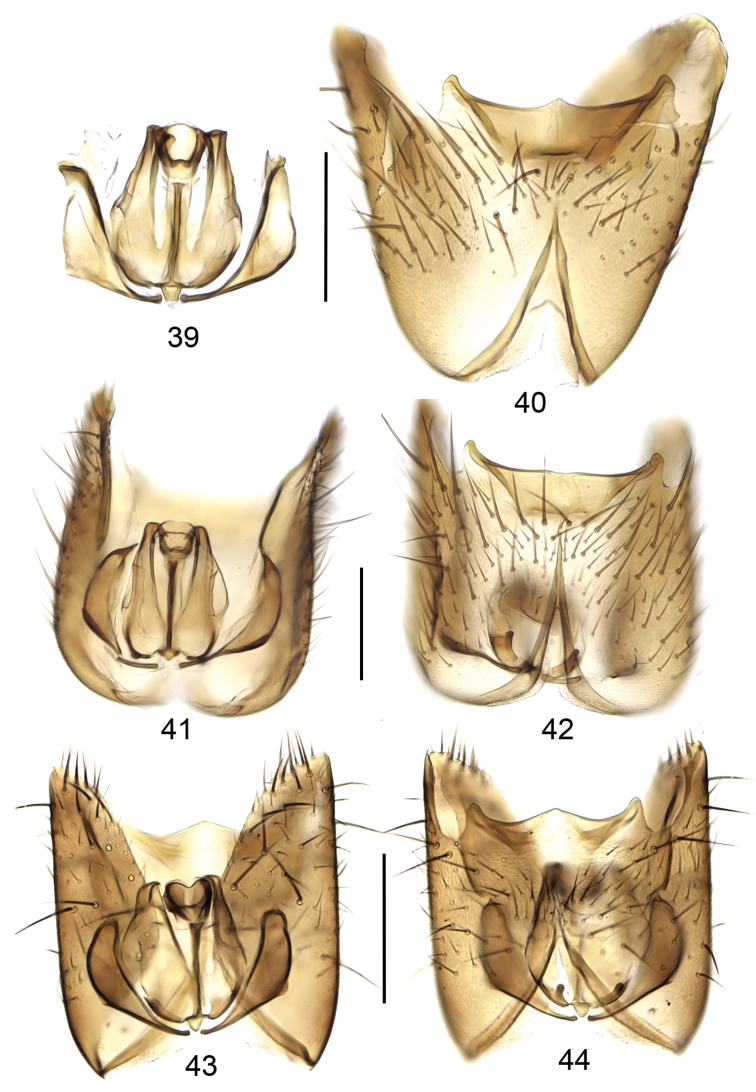
Gonocoxites with/and aedeagal complex, dorsal view (**39, 41, 43**) and ventral view (**40, 42, 44**). **39, 40** *Mycetophila evanida* **41, 42** *Mycetophila ruficollis* **43, 44** *Mycetophila sepulta*. Scale bar = 0.2 mm.

**Figures 45–50.**
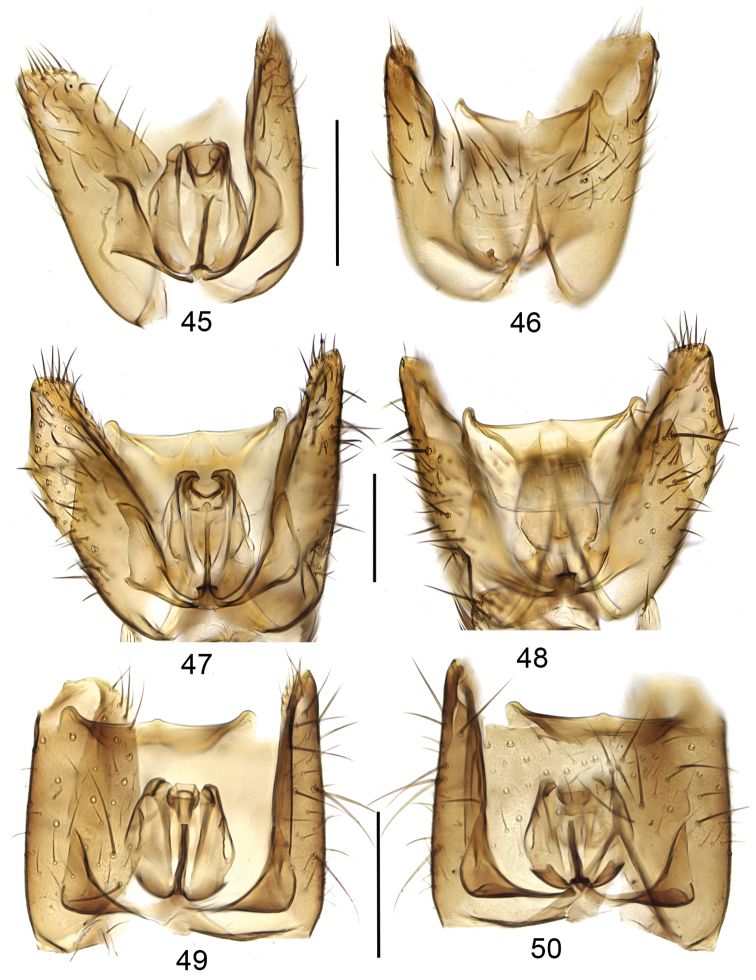
Gonocoxites with aedeagal complex, dorsal view (**45, 47, 49**) and ventral view (**46, 48, 50**). **45, 46** *Mycetophila strobli* **47, 48** *Mycetophila suffusala* **49, 50** *Mycetophila uninotata*. Scale bar = 0.2 mm.

## The species

### Mycetophila britannica

Taxon classificationAnimaliaDipteraMycetophilidae

Laštovka & Kidd, 1975

Figs 6 , 15 , 16 , 33 , 34

#### Material.

**ITALY**. 4♂♂, Sardinia, Alghero, near Nuraghe Palmavera, 40°35'N, 08°14'E, 63m, 21.xi.2005, sweeping, O. Kurina leg. (IZBE0200050, IZBE0200131, in alcohol with terminalia in glycerine; IZBE0200129, IZBE0200130, in alcohol, abdomen used for DNA sequence: SJ306, SJ313). **GREECE**. 1♂, Village Kerkini, Krousia Mts., 41°11'32,4"N, 23°03'59,5"E, 190m, 5.ix–11.ix.2007, Malaise trap, G. Ramel leg. (IZBE0200132, in alcohol, abdomen used for DNA sequence: SJ326); 1♂, Elodia, Cafe site, 41°12'46,8"N, 23°05'42,9"E, 10.iii-16.iii.2008, Malaise trap, G. Ramel leg. (IZBE0200134, in alcohol, abdomen used for DNA sequence: SJ17); 1♂, Village Kerkini, Cafe Elodia, 41°12'46,8"N, 023°05'42,9"E, 40m, 25.ii–2.iii.2008, Malaise trap, G. Ramel leg. (IZBE0200135, in alcohol, abdomen used for DNA sequence: SJ323); 1♂, Village Vironia, Beabies site, 41°19'15,4"N, 23°13'39,6"E, 1150m, 9.vi–15.vi.2008, Malaise trap, G. Ramel leg. (IZBE0200146, in alcohol with terminalia in glycerine, abdomen used for DNA sequence: SJ39); 1♂, Village Neo Petritsi, Sultanitsa site, 41°19'02,1"N, 23°12'05,0"E, 1485m, 30.vi-6.vii.2008, Malaise trap, G. Ramel leg. (IZBE0200136, in alcohol with terminalia in glycerine, abdomen used for DNA sequence: SJ9); 1♂, Village Neo Petritsi, Sultanitsa site, 41°19'02,1"N, 23°12'05,0"E, 1485m, 8.ix–14.ix.2008, Malaise trap, G. Ramel leg. (IZBE0200137, in alcohol, abdomen used for DNA sequence: SJ31); 1♂, Village Promohonas, Procom, 41°22'38,1"N, 023°21'58,8"E, 60m, 25.ii-2.iii.2008, malaise trap, G. Ramel leg. (IZBE0200138, in alcohol, abdomen used for DNA sequence: SJ324); 1♂, Village Neo Petritsi, Sultanitsa site, 41°19'02,1"N, 23°12'05,0"E, 1485m, 15.ix–21.ix.2008, Malaise trap, G. Ramel leg. (IZBE0200139, in alcohol, abdomen used for DNA sequence: SJ22); 1♂, Village Vironia, Ramna site, 41°17'42,5"N, 23°11'33,1"E, 750m, 17.xi–23.xi.2008, Malaise trap, G. Ramel leg. (IZBE0200140, in alcohol, abdomen used for DNA sequence: SJ29); 1♂, same as earlier, (IZBE0200141, in alcohol with terminalia in glycerine, abdomen used for DNA sequence: SJ40); 1♂, Village Vironia, Beabies site, 41°19'15,4"N, 23°13'39,6"E, 1150m, 15.ix-21.ix.2008, Malaise trap, G. Ramel leg. (IZBE0200142, in alcohol, abdomen used for DNA sequence: SJ14); 1♂, Village Vironia, Ramna site, 41°17'42,5"N, 23°11'33,1"E, 750m, 10.xi-16.xi.2008, Malaise trap, G. Ramel leg. (IZBE0200143, in alcohol, abdomen used for DNA sequence: SJ1); 1♂, Village Vironia, Ramna site, 41°17'42,5"N, 23°11'33,1"E, 750m, 8.xii–14.xii.2008, Malaise trap, G. Ramel leg. (IZBE0200144, in alcohol with terminalia in glycerine, abdomen used for DNA sequence: SJ36). **LEBANON**. 1♂, Kesrouane Mar Elias, 33°54'N, 35°32'E, 27.v–4.vi.2012, light trap, J. Kullberg leg. (IZBE0200145, in alcohol, abdomen used for DNA sequence: SJ315); 1♂, Kesrouane Mar Elias, 33°54'N, 35°32'E, 30.v.2012, light trap, J. Kullberg leg. (IZBE0200153, in alcohol, abdomen used for DNA sequence: SJ322).

#### Male terminalia.

Posterior margin of gonocoxite slightly concave ventromedially, and with abrupt and blunt projections laterally. Posterior impression wide. Anterior impression with anteriorly evenly divergent wide arms. Ventral branch of gonostylus with narrow, short and asymmetrical posterior process which bears minute warts; ventral surface with 6–9 long bristles deviating from other setosity; spine 1 and spine 2 of almost equal height and width; spine 1 sharply pointed; spine 2 blunt; spines 3 and 4 smaller, pointed and close to each other. Dorsal branch of gonostylus abruptly narrowed beyond the medial bristle; lateral margin with shallow concavity or almost straight; distal posterior process very small, separated from proximal posterior process by a narrow but distinct notch, both processes apically rounded. Posterior margin proximally from medial bristle with 5 gradually diminishing bristles followed by 2 small setae. Basal angle slightly rounded, basal margin with few setae. Distal posterior process apically with small seta, proximal posterior process apically bare. Ejaculatory apodeme with semi-rounded or proximally truncated base and without rim. Aedeagal guides wide, apically widened, extending beyond aedeagus distally, lateral impressions wide. Aedeagal apodemes laterally angular.

#### Intraspecific variation.

Laštovka and Kidd (1975) figured the ventromedial margin of the gonocoxite with a small convexity medially that was not observed in the studied material. They also described two different forms of the dorsal branch of the gonostylus: 1) slender and slightly narrowing beyond the medial bristle, and 2) shorter and abruptly narrowing beyond the medial bristle. The last character resembles that of *Mycetophila ruficollis*, *Mycetophila laffooni* Laštovka, 1972 and somewhat also *Mycetophila suffusala*. All studied specimens had the dorsal branch of the gonostylus slender, corresponding to the first form. Laštovka and Kidd (1975) found *Mycetophila britannica* to be most similar to *Mycetophila evanida*. In some studied specimens from Greece, the spine 2 on the ventral branch of the gonostylus is more massive, being longer than the spine 1. In Greek specimens the spine 2 in the ventral branch of the gonostylus is blunt while it is sharply pointed in Italian specimens.

#### Hosts and distribution.

*Mycetophila britannica* has been earlier reared from *Polyporus squamosus*, *Armillaria mellea*, *Hebeloma crustuliniforme*, *Russula nigricans*, *Hypholoma* sp. and *Lactarius resimus* (Laštovka and Kidd 1975, Yakovlev 1994, Chandler 2010), while we have studied sweepnetted and trapped material only. Having been described from the British Isles, the species is widely distributed in Western Europe extending also to Norway and the Middle East (Kjærandsen 2012, Chandler 2013). Except for a finding in Russian Karelia (Kjærandsen 2012) it is not found in Eastern Europe. According to Chandler and Ribeiro (1995) and Chandler et al. (2005), *Mycetophila britannica* is common in the Mediterranean region including Morocco.

### Mycetophila evanida

Taxon classificationAnimaliaDipteraMycetophilidae

Laštovka, 1972

Figs 8 , 21 , 22 , 39 , 40

#### Material.

**ESTONIA**. 1♂, Jõgeva county, Pataste, 58°34'52,2"N, 26°46'42,3"E, 5.x–19.x.2009, Malaise trap, J. Elts leg. (IZBE0200069, in alcohol with terminalia in glycerine, abdomen used for DNA sequence: SJ270); 1♂, Tartu county, Melliste, 58°19'43,8"N, 26°56'25,1"E, 4.x–18.x.2008, Malaise trap, O. Kurina leg. (IZBE0200070, in alcohol with terminalia in glycerine, abdomen used for DNA sequence: SJ230). **ITALY.** 1♂, Südtirol, N. Park Stilfser Joch, Unt. Tartscher Tal (S von Trafoi), 46°32'33,9"N, 10°30'17,2"E, 1630m, 27.vi-4.vii. 2005, Malaise trap, C. Lange and J. Ziegler leg. (IZBE0200071, in alcohol with terminalia in glycerine, abdomen used for DNA sequence: SJ53).

#### Male terminalia.

Posterior margin of gonocoxite ventromedially with clear convexity, and with abrupt and blunt projections laterally. Posterior impression rather wide. Anterior impression with anteriorly evenly divergent narrow arms. Ventral branch of gonostylus with semicircular posterior process which bears minute warts and few setae; spine 1 slender, evenly tapering and sharply pointed; spine 2 shorter, thicker and rather blunt; spines 3 and 4 much smaller than spine 1, sharply pointed and close to each other. Dorsal branch of gonostylus steeply tapering; lateral margin with shallow concavity; distal posterior process about half as high as proximal posterior process, both separated by shallow concavity. Distal posterior process with few setae; proximal posterior process bare with 2-3 setae deviating from other setosity on its base. Posterior margin proximally from medial bristle with 6 gradually diminishing bristles; internal surface with one somewhat stronger seta next to the medial bristle. Basal angle right-angled, basal margin with setae. Ejaculatory apodeme with subquadrate base and very wide rim. Aedeagus widened apically, apical margin slightly convex. Aedeagal guides with wide lateral impressions; apically narrow and rounded, not extending upper margin of aedeagus. Aedeagal apodemes laterally angular.

#### Intraspecific variation.

In some cases the base of the ejaculatory apodeme resembles *Mycetophila idonea*, because of having its basal margin somewhat concave. The holotype has an additional small spine on the ventral branch of the gonostylus close to spines 3 and 4 (cf. Laštovka (1972: fig. 12). In studied material this spine was not observed.

#### Hosts and distribution.

*Mycetophila evanida* has been reared from species of *Russula*, *Lactarius*, and *Tubaria* (Yakovlev 1994). The species is widely distributed in Europe extending also to the Eastern Palearctic (Laštovka 1972, Chandler 2013, Jakovlev 2014). The material from Estonia represents a new country record.

### Mycetophila ichneumonea

Taxon classificationAnimaliaDipteraMycetophilidae

Say, 1823

Figs 1 , 2 , 3 , 9 , 19 , 20 , 37 , 38

#### Material.

**FINLAND.** 1♂, Sodankylä, Syväkuru, 67°25'N, 026°35'E, 21.viii.2013, sweeping, J. Salmela leg. (IZBE0200150, in alcohol with terminalia in glycerine, abdomen used for DNA sequence: SJ434). **ESTONIA**. 1♂, Tartu county, Melliste, 58°20'N, 26°59'E, 20.viii–4.ix.2008, Malaise trap, O. Kurina leg. (IZBE0200073, in alcohol with terminalia in glycerine, abdomen used for DNA sequence: SJ272); 1♂, same as earlier except 24.x–16.xi.2008 (IZBE0200074, in alcohol with terminalia in glycerine, abdomen used for DNA sequence: SJ226); 1♂, Saare county, Orissaare, 58°33'19"N, 23°05'12"E, 18.x–5.xi.2008, Malaise trap, H. Jäe leg. (IZBE0200075, in alcohol, abdomen used for DNA sequence: SJ239); 1♂, Tartu county, Maiorg near Annikoru, 58°16'41,6"N, 26°20'03,6"E, 1.v–16.v.2009, Malaise trap, O. Kurina leg. (IZBE0200076, in alcohol with terminalia in glycerine, abdomen used for DNA sequence: SJ129); 1♂, Jõgeva county, Kaiu, 58°39'20,4"N, 26°52'43,2"E, reared from *Cortinarius traganus*, coll. 21.ix.2011, emerg. 24.x.2011, S. Jürgenstein leg. (IZBE0200077, in alcohol); 1♂, Tartu county, Järvselja, 58°17'45"N, 27°15'41,7"E, reared from *Lactarius helvus*, coll. 23.ix.2011, emerg. 12.x.2011, S. Jürgenstein leg. (IZBE0200078, in alcohol, abdomen used for DNA sequence: SJ261); 1♂, Tartu county, Järvselja, 58°17'45"N, 27°15'41,7"E, reared from *Lactarius torminosus*, coll. 23.ix.2011, emerg. 10.x.2011, S. Jürgenstein leg. (IZBE0200079, in alcohol, abdomen used for DNA sequence: SJ223); 1♂, Põlva county, Viira, 58°0'N, 27°11'E, reared from *Cortinarius hemitrichus*, coll. 10.x.2012, emerg. 31.x.2012, S. Jürgenstein leg. (IZBE0200081, in alcohol, abdomen used for DNA sequence: SJ335); 1♂, Jõgeva county, Kaiu, 58°39'20,4"N, 26°52'43,2"E, reared from *Cortinarius armeniacus*, coll. 1.x.2012, emerg. 29.x.2012, S. Jürgenstein leg. (IZBE0200082, in alcohol, abdomen used for DNA sequence: SJ337); 1♂, Jõgeva county, Kaiu, 58°39'20,4"N, 26°52'43,2"E, reared from *Cortinarius depressus*, coll. 1.x.2012, emerg. 31.x.2012, S. Jürgenstein leg. (IZBE0200083, in alcohol, abdomen used for DNA sequence: SJ338); 1♂, Lääne county, Haapsalu, 58°57'N, 23°32'E, reared from *Cortinarius saturninus*, coll. 25.ix.2012, emerg. 22.x.2012, S. Jürgenstein leg. (IZBE0200085, in alcohol, abdomen used for DNA sequence: SJ342); 1♂, Võru county, Rõuge, 57°44'N, 26°55'E, reared from Cortinarius subgenus Telamonia, coll. 3.ix.2012, emerg. 21.ix.2012, S. Jürgenstein leg. (IZBE0200086, in alcohol, abdomen used for DNA sequence: SJ341); 1♂, Tartu county, Uniküla, 58°16'N, 26°55'E, reared from *Cortinarius dolabratus*, coll. 2.x.2012, emerg. 2.xi.2012, S. Jürgenstein leg. (IZBE0200087, in alcohol, abdomen used for DNA sequence: SJ345); 1♂, Jõgeva county, Kõduküla, 58°34'N, 26°31'E, reared from *Lactarius deterrimus*, coll. 15.ix.2012, emerg. 2.x.2012, S. Jürgenstein leg. (IZBE0200088, in alcohol, abdomen used for DNA sequence: SJ346); 1♂, Valga county, Miti, 58°06'16,7"N, 26°22'28,9"E, reared from *Lactarius rufus*, coll. 14.ix.2011, emerg. 3.x.2011, S. Jürgenstein leg. (IZBE0200089, in alcohol with terminalia in glycerine); 1♂, Valga county, Soontaga, 58°01'42,6"N, 26°04'29,3"E, reared from *Lactarius rufus*, coll. 16.ix.2011, emerg. 12.x.2011, S. Jürgenstein leg. (IZBE0200090, in alcohol); 1♂, Valga county, Soontaga, 58°01'42,6"N, 26°04'29,3"E, reared from *Lactarius rufus*, coll. 16.ix.2011, emerg. 3.x.2011, S. Jürgenstein leg. (IZBE0200091, in alcohol); 1♂, Valga county, Soontaga, 58°01'42,6"N, 26°04'29,3"E, reared from *Lactarius rufus*, coll. 16.ix.2011, emerg. 6.x.2011, S. Jürgenstein leg. (IZBE0200152, in alcohol); 1♂, Valga county, Soontaga, 58°01'42,6"N, 26°04'29,3"E, reared from *Lactarius camphoratus*, coll. 16.ix.2011, emerg. 5.x.2011, S. Jürgenstein leg. (IZBE0200092, in alcohol); 1♂, Põlva county, Ihamaru, 58°06'00,40"N, 26°55'55,45"E, reared from *Lactarius rufus*, coll. 18.ix.2011, emerg. 21.x.2011, S. Jürgenstein leg. (IZBE0200093, in alcohol); 1♂, Põlva county, Ihamaru, 58°06'00,40"N, 26°55'55, 45"E, reared from *Rhodocollybia butyracea*, coll. 18.ix.2011, emerg. 5.x.2011, S. Jürgenstein leg. (IZBE0200094, in alcohol); 1♂, Jõgeva county, Kaiu, 58°39'20,4’’ N, 26°52'43,2"E, reared from *Cortinarius* sp., coll. 21.ix.2011, emerg. 17.x.2011, S. Jürgenstein leg. (IZBE0200095, in alcohol); 1♂, Jõgeva county, Kaiu, 58°39'20,4"N, 26°52'43,2"E, reared from *Cortinarius alborufescens*, coll. 21.ix.2011, emerg. 5.x.2011, S. Jürgenstein leg. (IZBE0200096, in alcohol); 1♂, Valga county, Soontaga, 58°01'42,6"N, 26°04'29,3"E, reared from *Lactarius helvus*, coll. 16.ix.2011, emerg. 6.x.2011, S. Jürgenstein leg. (IZBE0200097, in alcohol); 1♂, Jõgeva county, Kaiu, 58°39'20,4"N, 26°52'43,2"E, reared from *Cortinarius* sp., coll. 21.ix.2011, emerg. 12.x.2011, S. Jürgenstein leg. (IZBE0200098, in alcohol); 1♂, Valga county, Soontaga, 58°01'42,6"N, 26°04'29,3"E, reared from *Lactarius helvus*, coll. 16.ix.2011, emerg. 12.x.2011, S. Jürgenstein leg. (IZBE0200099, in alcohol); 1♂, Valga county, Soontaga, 58°01'42,6"N, 26°04'29,3"E, reared from *Lactarius rufus*, coll. 16.ix.2011, emerg. 10.x.2011, S. Jürgenstein leg. (IZBE0200101, in alcohol with terminalia in glycerine); 1♂, Valga county, Soontaga, 58°01'42,6"N, 26°04'29,3"E, reared from *Lactarius rufus*, coll. 16.ix.2011, emerg. 6.x.2011, S. Jürgenstein leg. (IZBE0200102, in alcohol); 1♂, Valga county, Soontaga, 58°01'42,6"N, 26°04'29,3"E, reared from *Lactarius rufus*, coll. 16.ix.2011, emerg. 12.x.2011, S. Jürgenstein leg. (IZBE0200103, in alcohol); 1♂, Võru county, Rõuge, 57°44'N, 26°55'E, reared from *Cortinarius alborufesens*, coll. 3.ix.2012, emerg. 24.ix.2012, S. Jürgenstein leg. (IZBE0200104, in alcohol, abdomen used for DNA sequence: SJ344); 1♂, Lääne county, Haapsalu, 58°57'N, 23°32'E, reared from *Cortinarius cotoneus*, coll. 25.ix.2012, emerg. 17.x.2012, S. Jürgenstein leg. (IZBE0200105, in alcohol with terminalia in glycerine, abdomen used for DNA sequence: SJ343); 1♂, Lääne county, Vormsi 59°0'N, 23°15'E, reared from *Russula vinosa*, coll. 25.viii.1991, emerg. 7. ix.1991, O. Kurina leg. (IZBE0200106, pinned); 1♂, Pärnu county, Nigula NR, 58°0'41"N, 24°40'60"E, reared from *Megacollybia platyphylla*, coll. 5.viii.1990, emerg. 17.viii.1990, O. Kurina leg. (IZBE0200107, pinned); 1♂, Valga county, Soontaga, 58°01'42,6"N, 26°04'29,3"E, reared from *Lactarius helvus*, coll. 16.ix.2011, emerg. 12.x.2011, S. Jürgenstein leg. (IZBE0200108, in alcohol); 1♂, Põlva county, Ihamaru, 58°06'00,40"N, 26°55'55,45"E, reared from *Lactarius rufus*, coll. 18.ix.2011, emerg. 17.x.2011, S. Jürgenstein leg. (IZBE0200109, in alcohol); 1♂, Valga county, Soontaga, 58°01'42,6"N, 26°04'29,3"E, reared from *Lactarius helvus*, coll. 16.ix.2011, emerg. 5.x.2011, S. Jürgenstein leg. (IZBE0200033, in alcohol, abdomen used for DNA sequence: SJ147; published earlier by Põldmaa et al. 2015); 1♂, Põlva county, Ihamaru, 58°06'00,40"N, 26°55'55, 45"E, reared from *Lactarius camphoratus*, coll. 18.ix.2011, emerg. 19.x.2011, S. Jürgenstein leg. (IZBE0200156, in alcohol, abdomen used for DNA sequence: SJ138); 1♂, Tartu county, Järvselja, 58°17'45"N, 27°15'41,7"E, reared from *Cortinarius brunneus*, coll. 23.ix.2011, emerg. 10.x.2011, S. Jürgenstein leg. (IZBE0200157, in alcohol, abdomen used for DNA sequence: SJ148). **SLOVAKIA**. 1♂, NP Slovensky kras, Silickà Ladnica, 48°33'00,0"N, 020°30'14,4"E, 505m, 3.vi.2009, sweeping, O. Kurina leg. (IZBE0200111, in alcohol with terminalia in glycerine, abdomen used for DNA sequence: SJ318). **GEORGIA**. 1♂, Surami, 42°01'34,2"N, 43°29'52,5"E, 941m, 18.v.2012, sweeping, O. Kurina leg. (IZBE0200112, in alcohol with terminalia in glycerine, abdomen used for DNA sequence: SJ66); 1♂, Bakuriani, 41°45'46,2"N, 43°30'16,7"E, 1626m, 31.viii.2014, sweeping, O. Kurina leg. (IZBE0200242, slide mounted in Euparal); 1♂, same as earlier except 1.ix.2014 (IZBE0200243, in alcohol); 3♂♂, Bakuriani 1.2 km-W, 41°44'13,0"N, 43°30'45,1"E, 1741m, 1.ix.2014, sweeping, O. Kurina leg. (IZBE0200244–IZBE0200246, in alcohol).

#### Male terminalia.

Posterior margin of gonocoxite ventromedially with shallow convexity, and with abrupt and blunt projections laterally. Posterior impression with narrow base and well widened posterior part. Anterior impression with anteriorly evenly divergent narrow arms. Ventral branch of gonostylus with symmetrical semi-oval posterior process, which bears minute warts and few setae on ventral surface; spine 1 wide, sharply pointed; spine 2 similar or somewhat longer but more slender; spines 3 and 4 about twice as small, evenly tapering and with about equal distance between each other and spine 2. Ventral branch of gonostylus with 4–5 strong bristles deviating from other setosity laterally on ventral surface. Dorsal branch of gonostylus steeply tapering; lateral margin with deep concavity; proximal posterior process about twice as high as distal posterior process, both separated by deep concavity. Distal posterior process with apical small setula and with a basal strong seta deviating from other setosity; proximal posterior process apically rounded with 1–2 strong basal setae deviating from other setosity. Posterior margin proximally from medial bristle with 4–5 gradually diminishing bristles followed by 3–4 smaller setae; internal surface with a stronger seta next to the medial bristle. Basal angle clearly outlined, angular or somewhat rounded; basal margin with few setae. Ejaculatory apodeme with campanulate base and without rim. Aedeagus oval or cross shaped. Aedeagal guides extending over apical part of aedeagus; apically rounded and divided into two lamellae; with lateral impressions very small. Aedeagal apodemes laterally slightly angular or arched and pointed apically.

#### Intraspecific variation.

In some cases the lateral margin of the dorsal branch of the gonostylus is shallower, and the distal posterior process and the posterior margin of the dorsal branch of the gonostylus resemble those of *Mycetophila uninotata*. The spine 2 on the ventral branch of the gonostylus compared to the spine 1 can be more prominent than described by Laštovka (1972).

#### Hosts and distribution.

Known from many species of Agaricales and Russulales (Kurina 1991, Yakovlev 1994, Chandler 2010, Ševčík 2010). Our material from pine dominated boreal forests in Estonia indicates specialisation to *Lactarius* (Russulales) and *Cortinarius* (Agaricales). Widely distributed in Europe extending also to the Eastern Palaearctic, the Middle East and the Nearctic region (Laštovka 1972, Chandler 2013). The material from Georgia represents a new country record.

### Mycetophila idonea

Taxon classificationAnimaliaDipteraMycetophilidae

Laštovka, 1972

Figs 7 , 17 , 18 , 35 , 36

#### Material.

**ESTONIA.** 1♂, Valga county, Soontaga, 58°01'42,6"N, 26°04'29,3"E, reared from *Amanita citrina*, coll. 17.ix.2013, emerg. 2.x.2013, S. Jürgenstein leg. (IZBE0200158, in alcohol with terminalia in glycerine, abdomen used for DNA sequence: SJ386). **POLAND**. 2♂♂, Białowieża 2 km SW, village Gródek, 52°41'02,8"N, 23°49'33,1"E, 17.viii.2007, sweeping, O. Kurina leg. (IZBE0200027, IZBE0200035, in alcohol with terminalia in glycerine, abdomen used for DNA sequence: SJ54, SJ56). **SLOVAKIA**. 1♂, Muranska planina, near Klak, 48°46'53"N, 019°59'21,3"E, 1211m, 28.v.2009, sweeping, O. Kurina leg. (IZBE0200047, in alcohol with terminalia in glycerine, abdomen used for DNA sequence: SJ317). **GEORGIA**. 1♂, Dgnali NE of Zhinvali, 42°13'25,9"N, 44°40'12,1"E, 914m, 15.v.2012, sweeping, O. Kurina leg. (IZBE0200055, in alcohol with terminalia in glycerine, abdomen used for DNA sequence: SJ69); 2♂♂, Marelisi SW of Surami, 41°57'56,0"N, 43°17'20,7"E, 412m, 19.v.2012, sweeping, O. Kurina leg. (IZBE0200056, IZBE0200057, in alcohol with terminalia in glycerine, abdomen used for DNA sequence: SJ72, SJ78); 1♂, Kintrishi NP, 41°45'11,7"N, 041°58'38,4"E, 453m, 21.v.2013, sweeping, O. Kurina leg. (IZBE0200058, in alcohol, abdomen used for DNA sequence: SJ327); 1♂, Kintrishi NP, 41°45'11,7"N, 041°58'38,4"E, 453m, 22.v.2013, sweeping, O. Kurina leg. (IZBE0200149, in alcohol, abdomen used for DNA sequence: SJ316); 1♂, Saguramo N of Tbilisi, 41°53'04,3"N, 44°46'46,5"E, 915m, 15.v.2012, sweeping, O. Kurina leg. (IZBE0200059, in alcohol with terminalia in glycerine, abdomen used for DNA sequence: SJ64); 1♂, same as earlier, (IZBE0200060, in alcohol with terminalia in glycerin); 1♂, Saguramo N of Tbilisi, 41°53'08,0"N, 44°46'44,2"E, 889m, 4.ix.2014, sweeping, O. Kurina leg. (IZBE0200237, slide mounted in Euparal); 1♂, Lagotekhi, 41°49'N, 46°17'E, 15.vi–25.vi.2014, Malaise trap, G. Japoshvili leg. (IZBE0200238, in alcohol with terminalia slide mounted in Euparal).

#### Male terminalia.

Posterior margin of gonocoxite with slight convexity ventromedially, and with abrupt and blunt projections laterally. Posterior impression with considerably narrow base and widened posterior part. Anterior impression with narrow, anteriorly evenly divergent arms. Ventral branch of gonostylus with posterior process wide and shallow (asymmetrical), with minute warts; spine 1 and spine 2 almost the same high; spine 1 slender, evenly tapering and sharply pointed; spine 2 thicker and blunt; spines 3 and 4 smaller, blunt, with about equal distance between each other and spine 2. Dorsal branch of gonostylus evenly tapering, lateral margin without concavity; distal posterior process about half as high as proximal posterior process, both separated by deep concavity. Distal posterior process bears apical and subapical setae, proximal posterior process bare and apically rounded. Posterior margin proximally from medial bristle with 3-4 gradually diminishing bristles; internal surface with one somewhat stronger seta next to the medial bristle; otherwise the setosity has no special arrangement. Basal angle slightly rounded, basal margin with few setae. Ejaculatory apodeme with concave base and narrow rim. Aedeagus widened apically and truncated. Aedeagal guides with well outlined, wide lateral impressions; apically widened and rounded covering edges of aedeagus. Aedeagal apodemes laterally angular.

#### Intraspecific variation.

In some specimens from Georgia and Poland the base of the ejaculatory apodeme is blunt, resembling that of *Mycetophila strobli*. In a few cases the spine 2 on the ventral branch of the gonostylus is slender.

#### Hosts and distribution.

*Mycetophila idonea* has been reared from about 65 species of Agaricales and Russulales and also from *Boletus impolitus* (Yakovlev, 1994). The species is widely recorded from Europe extending to the Middle East and Eastern Palaearctic (Chandler 2013). The species was erroneously reported as overwintering in Estonian caves by Kurina (1996); after critical study, the material was found to belong to *Mycetophila uninotata*. However, the occurrence in Estonia is confirmed by a new rearing record presented herein. The material from Georgia represents a new country record.

### Mycetophila ruficollis

Taxon classificationAnimaliaDipteraMycetophilidae

Meigen, 1818

Figs 10 , 23 , 24 , 41 , 42

#### Material.

**POLAND**. 1♂, Białowieża 2 km SW, Gródek, 52°41'02,8"N, 23°49'33,1"E, 17.viii.2007, sweeping, O. Kurina leg. (IZBE0200119, in alcohol with terminalia in glycerine, abdomen used for DNA sequence: SJ55). **ESTONIA**. 1♂, Tartu county, Maiorg near Annikoru, 58°16'41,6"N, 26°20'03,6"E, 17.ix.–2.x.2008, Malaise trap, O. Kurina leg. (IZBE0200120, in alcohol with terminalia in glycerine, abdomen used for DNA sequence: SJ229); 1♂, Jõgeva county, Pataste, 58°34'52,2"N, 26°46'42,3"E, 18.x–30.x.2008, Malaise trap, J. Elts leg. (IZBE0200121, in alcohol with terminalia in glycerine, abdomen used for DNA sequence: SJ268); 1♂, Lääne county, Oonga, 58°0'41"N, 24°40'60"E, reared from *Armillaria mellea*, coll. 8.ix.1994, emerg. 21.ix.1994, O. Kurina leg. (IZBE0200123, pinned); 1♂, Saare county, Abruka, 58°9'50"N, 22°30'14"E, reared from *Megacollybia platyphylla*, coll. 11.ix.1991, emerg. 27.ix.1991, O. Kurina leg. (IZBE0200124, pinned); 1♂, Tartu county, Järvselja, 58°17'45"N, 27°15'41,7"E, reared from *Pholiota aurivella*, coll. 4.ix.1989, emerg. 25.ix.1989, O. Kurina leg. (IZBE0200125, pinned); 1♂, Tartu county, Järvselja, 58°17'45"N, 27°15'41,7"E, reared from *Entoloma* sp., coll. 27.viii.1989, emerg. 11.ix.1989, O. Kurina leg. (IZBE0200126, pinned); 1♂, Jõgeva county, Kõduküla, 58°34'N, 26°31'E, reared from *Rhodocollybia butyracea*, coll. 15.ix.2012, emerg. 2.x.2012, S. Jürgenstein leg. (IZBE0200127, in alcohol, abdomen used for DNA sequence: SJ339); 1♂, Saare county, Abruka, 58°9'50"N, 22°30'14"E, 21.ix.2013 reared from *Armillaria borealis*, coll. 21.ix.2013, emerg. 4.x.2013, O. Kurina leg. (IZBE0200128, in alcohol with terminalia in glycerine, abdomen used for DNA sequence: SJ382). **GEORGIA**. 1♂, Lagotekhi, 41°49'N, 46°17'E, 15.vi–25.vi.2014, Malaise trap, G. Japoshvili leg. (IZBE0200247, in alcohol).

#### Male terminalia.

Posterior margin of gonocoxite undulate or slightly concave ventromedially, and with abrupt and blunt projections laterally. Posterior impression wide and uncompressed. Anterior impression with wide arms which are abruptly divergent anteriorly. Ventral branch of gonostylus with short and semicircular posterior process, with minute warts; ventral surface with long and slender bristles deviating from other setosity; spine 1 slender, sharply pointed, about half as wide as spine 2; spine 2 blunt, about half as high as spine 1; spines 3 and 4 smaller, sharply pointed, close to each other. Dorsal branch of gonostylus abruptly narrowed beyond the medial bristle. Posterior margin proximally from medial bristle with 7–9 gradually diminishing bristles; internal surface with a stronger seta next to the medial bristle. Lateral margin without concavity, almost straight. Basal angle slightly rounded, basal margin with few setae. Distal posterior process very shallow, almost unnoticeable; proximal posterior process high and massive, apically rounded. Distal posterior process with setae, proximal posterior process apically bare. Dorsal surface with a distinct band of setae from base of posterior processes to basal angle. Ejaculatory apodeme proximally narrows, with campanulate base and narrow rim. Aedeagal guides wide, apically widened and rounded, not extending beyond aedeagus distally; lateral impressions wide. Aedeagal apodemes laterally angular.

#### Intraspecific variation.

The combination of wide 4^th^ and considerably short 5^th^ palpal segments, the ventroapical margin of the gonocoxite without any medial projections and the apically abruptly narrowed dorsal branch of the gonostylus are unique among European species of the group.

#### Hosts and distribution.

*Mycetophila ruficollis* is reared from 35 species of macrofungi (Yakovlev 1994). However, some of the rearing records in the literature may possibly refer to entire group: e.g. records from *Lactarius* and *Russul* a by Ribeiro (1990) and some records in Yakovlev (1994). Our records reveal saprotrophic members of the Agaricales as the host of this species. The species is widely distributed in Europe (Chandler 2013, Jakovlev 2014, Kjærandsen 2012) extending also to the Middle East and Eastern Palaearctic (Chandler 2013). The material from Georgia represents a new country record.

### Mycetophila sepulta

Taxon classificationAnimaliaDipteraMycetophilidae

Laffoon, 1957

Figs 11 , 25 , 26 , 43 , 44

#### Material.

**UNITED KINGDOM**. 1♂, Berks, California, Country Park, 1.xi.2001, sweeping, P. J. Chandler leg. (PCPC, pinned, terminalia slide mounted in Euparal); 1♂, Oxon, Spartum Fen, 15.x.1999, sweeping, P. J. Chandler leg. (PCPC, pinned, terminalia slide mounted in Euparal).

#### Male terminalia.

Posterior margin of gonocoxite with clear convexity ventromedially, and with abrupt and blunt projections laterally. Posterior impression considerably narrow at base but well widening posteriorly. Anterior impression with anteriorly evenly divergent narrow arms. Ventral branch of gonostylus with posterior process narrow, asymmetrical and high, with minute warts; spine 1 very slender and sharply pointed; spine 2 somewhat wider than spine 1, but also pointed and of same length; spines 3 and 4 smaller, pointed and rather close to each other. Dorsal branch of gonostylus slightly tapering, somewhat constricted at the medial bristle; lateral margin without or with very shallow concavity; distal posterior process and proximal posterior process almost of same height, apically rounded, separated by a rather wide notch. Distal posterior process with apical seta, proximal posterior process subapically with 3 setae. Posterior margin proximally from medial bristle with 4-5 gradually diminishing bristles followed by 2-3 setae; internal surface with a stronger seta next to the medial bristle. Basal angle slightly rounded, basal margin with few setae. Ejaculatory apodeme with heart-shaped base and with very narrow or barely visible rim. Aedeagus apically concave. Aedeagal guides rather wide, apically widened, not extending beyond aedeagus distally, lateral impressions wide. Aedeagal apodemes laterally angular.

#### Intraspecific variation.

In comparison with figures by Laštovka and Kidd (1975: fig. 30), the studied specimens have the distal posterior process of the dorsal branch of the gonostylus higher and the notch between the processes more clearly outlined. Laffoon (1957) mentioned *Mycetophila sepulta* to be closely allied to *Mycetophila ichneumonea*, *Mycetophila ruficollis* and *Mycetophila parvimaculata* Van Duzee, 1928.

#### Hosts and distribution.

The species, described from North America, has rather scattered distribution in Western Europe, extending to Sweden (Chandler 2013, Kjærandsen 2012). In North America, it has a wide distribution from Alaska to California and Texas (Laffoon 1957). The only rearing record is that by Laštovka and Kidd (1975) from *Hypholoma elongatum*.

### Mycetophila strobli

Taxon classificationAnimaliaDipteraMycetophilidae

Laštovka, 1972

Figs 12 , 27 , 28 , 45 , 46

#### Material.

**NORWAY**. 1♂, Troms, Svensby, 69°40'01,2"N, 019°49'58,8"E, 18.vii.2008, sweeping, O. Kurina leg. (IZBE0200061, in alcohol with terminalia in glycerine, abdomen used for DNA sequence: SJ305). **ESTONIA**. 1♂, Tartu county, Vapramäe, 58°15'9,28"N, 26°27'46,1"E, reared from *Russula delica* (No 1057), coll. 8.ix.1995, emerg. 21.ix.1995, O. Kurina leg. (IZBE0200239, pinned with terminalia in Euparal); 1♂, Pärnu county, Nigula NR, 58°9'N, 24°58'E, reared from *Lactarius torminosus*, coll. 22.viii.1993, emerg. 2.ix.1993, O. Kurina leg. (IZBE0200240, pinned with terminalia in glycerine); 2♂♂, Tartu county, Mustametsa, Välgi, 58°36'53,09"N, 26°53'56,1"E, reared from *Russula delica*, coll. 1.x.2013, emerg. 14.x.2013 and 16.x.2014, S. Jürgenstein leg. (IZBE0200062, IZBE0200063, in alcohol with terminalia in glycerine, abdomen used for DNA sequence: SJ379, SJ380); 1♂, Saare county, Muhu, Igaküla, 58°36'3,5"N, 23°07'42"E, 4.x–18.x.2008, Malaise trap, H. Jäe leg. (IZBE0200064, in alcohol with terminalia in glycerine, abdomen used for DNA sequence: SJ224); 1♂, Jõgeva county, Kaiu, 58°39'20,4"N, 26°52'43,2"E, reared from *Russula nigricans*, coll. 25.ix.2013, emerg. 7.x.2013, S. Jürgenstein leg. (IZBE0200065, in alcohol with terminalia in glycerine, abdomen used for DNA sequence: SJ385); 1♂, Põlva county, Palojärv, 58°2'57,3"N, 27°7'35,73"E, reared from *Russula adusta*, coll. 01.x.2013, emerg. 7.x.2013, S. Jürgenstein leg. (IZBE0200066, in alcohol with terminalia in glycerine, abdomen used for DNA sequence: SJ383); 1♂, Rapla county, Vardi, 59°1'27,07"N, 24°26'28,9"E, reared from *Lactarius deterrimus*, coll. 1.xi.2012, emerg. 5.xi.2012, S. Jürgenstein leg. (IZBE0200067, in alcohol, abdomen used for DNA sequence: SJ340). **ITALY**. 1♂, Südtirol, N. Park Stilfser Joch, Schmelz (SW von Prad), 46°36'42,1"N, 10°34'35,6"E, 940m, 15.viii–24.viii.2005, Malaise trap, C. Lange and J. Ziegler leg. (IZBE0200068, in alcohol with terminalia in glycerine, abdomen used for DNA sequence: SJ57).

#### Male terminalia.

Posterior margin of gonocoxite ventromedially with clear angular convexity, and with abrupt and blunt projections laterally. Posterior impression with narrow base and well widened posterior part. Anterior impression with divergent arms which are sinuate at anterior fourth. Ventral branch of gonostylus with asymmetrical posterior process which bears minute warts and few setae; spine 1 short and sharply pointed; spine 2 blunt, almost twice as high and thick as spine 1; spines 3 and 4 sharply pointed, close to each other and similar in size to spine 1. Ventral branch of gonostylus with a few strong bristles deviating from other setosity laterally on ventral surface. Dorsal branch of gonostylus slightly tapering, lateral margin with shallow concavity; proximal posterior process about three times as high as distal posterior process, both separated with a shallow concavity. Distal posterior process apically and subapically with few setae; proximal posterior process bare and apically rounded. Posterior margin proximally from medial bristle with 4-6 gradually diminishing bristles; internal surface with one somewhat stronger seta next to the medial bristle. Basal angle slightly rounded, basal margin bare. Ejaculatory apodeme with semi-oval base and wide rim. Aedeagus slightly widened apically and with apical margin convex. Aedeagal guides with wide lateral impressions; subapically constricted and apically rounded, not extending upper margin of aedeagus. Aedeagal apodemes laterally angular.

#### Intraspecific variation.

Occasionally the spine 2 on the ventral branch of the gonostylus is somewhat slender and pointed.

#### Hosts and distribution.

*Mycetophila strobli* has been reared from species of *Russula*, *Lactarius*, *Suillus*, *Collybia*, *Armillaria*, *Kuehneromyces* and *Cortinarius* (Yakovlev 1994, original data). The species is widely distributed in Europe extending to the Middle East (Chandler 2013). The material from Norway represents a new country record.

### Mycetophila suffusala

Taxon classificationAnimaliaDipteraMycetophilidae

Chandler & Ribeiro, 1995

Figs 13 , 29 , 30 , 47 , 48

#### Material.

**PORTUGAL**. 2♂♂, **Madeira**, Queimadas, 10.ix–11.ix.1986, P. Ohm leg. (PCPC, pinned with terminalia in Euparal). **SPAIN**. 2 ♂♂, **Tenerife**, near top of west ridge at Izaña, 2350m a.s.l., 29.iii.1984, N.P. Ashmole leg. (PCPC, pinned with terminalia in Euparal).

#### Male terminalia.

Posterior margin of gonocoxite ventrally straight except for diminutive central prominence, which is somewhat sunken into the posterior impression, and with abrupt and blunt projections laterally. Posterior impression wide and uncompressed. Anterior impression with evenly divergent arms anteriorly. Ventral branch of gonostylus with posterior process wide and angular, with minute warts; ventral surface with long and slender bristles deviating from other setosity; spine 1 sharply pointed, about as wide as spine 2; spine 2 geniculate, blunt, about as high as spine 1; spines 3 and 4 smaller, sharply pointed, close to each other. Dorsal branch of gonostylus abruptly narrowed beyond the medial bristle; lateral margin with concavity; distal posterior process very shallow, almost unnoticeable; proximal posterior process high and massive, apically rounded. Distal posterior process with setae, proximal posterior process apically bare. Dorsal surface with an indistinct band of setae from base of posterior processes to basal angle; the setae near basal angle are deviating from other setosity. Posterior margin proximally from medial bristle with 10–13 gradually diminishing bristles; internal surface with a stronger seta next to the medial bristle. Basal angle slightly rounded, basal margin with few setae. Ejaculatory apodeme with rectangular base and without rim. Aedeagus obovoid, with apical concavity. Aedeagal guides wide, apically hooked, extending beyond aedeagus distally, lateral impressions wide. Aedeagal apodemes laterally angular.

#### Intraspecific variation.

Because of its larger size and details of the maxillary palpus, the species resembles *Mycetophila ruficollis* and by general structure of the male terminalia also *Mycetophila britannica*. However, the dark apical shade on the wing and details of the terminalia allow it to be safely distinguished.

#### Hosts and distribution.

So far recorded only from Madeira and the Canary Islands (Chandler and Ribeiro 1995). Hosts unknown.

### Mycetophila uninotata

Taxon classificationAnimaliaDipteraMycetophilidae

Zetterstedt, 1852

Figs 14 , 31 , 32 , 49 , 50

#### Material.

**NORWAY**. 2♂♂, Troms, Svensby, 69°40'01,2"N, 019°49'58,8"E, 18.vii. 2008, sweeping, O. Kurina leg. (IZBE0200113, IZBE0200072, in alcohol with terminalia in glycerine, abdomen used for DNA sequence: SJ303, SJ304). **FINLAND**. 3♂♂, Sodankylä, Paistipuolet, 75°319'15"N, 34°66'98,8"E, 1.vi–29.vi.2009, sweeping, J. Salmela leg. (IZBE0200114, IZBE0200115, IZBE0200116, in alcohol with terminalia in glycerine, abdomen used for DNA sequence: SJ436, SJ438, SJ439). **ESTONIA**. 1♂, Põlva county, Piusa cave, 57°54'N, 27°28'E, 1.ii.1996, sweeping, O. Kurina leg. (IZBE0200241, pinned with terminalia in glycerine); 1♂, Jõgeva county, Pataste, 58°34'52,2"N, 26°46'42,3"E, 10.ix–20.ix.2008, Malaise trap, J. Elts leg. (IZBE0200151, in alcohol with terminalia in glycerine, abdomen used for DNA sequence: SJ243); 1♂, Harju county, Üksnurme, 59°17'42,5"N, 24°37'41,1"E, 22.ix-12.x.2008, Malaise trap, E. Ilumäe leg. (IZBE0200117, in alcohol with terminalia in glycerine; abdomen used for DNA sequence: SJ227). **SLOVAKIA**. 1♂, NP Slovensky kras, Silickà Ladnica, 48°33'00,0"N, 020°30'14,4"E, 505m, 4.vi.2009, sweeping, O. Kurina leg. (IZBE0200118, in alcohol, abdomen used for DNA sequence: SJ320).

#### Male terminalia.

Posterior margin of gonocoxite with shallow convexity ventromedially, and with blunt and oblique projections laterally. Posterior impression very wide and compressed, with emarginated anterior margin. Anterior impression with anteriorly evenly divergent narrow arms. Ventral branch of gonostylus with asymmetrical, narrow and elongated posterior process with dense and long warts; spine 1 sharply pointed; spine 2 about the same size, pointed; spines 3 and 4 smaller, pointed, closer to each other than to spine 2. Dorsal branch of gonostylus steeply tapering; lateral margin with shallow concavity; distal posterior process and proximal posterior process about the same height, both separated by a deep notch. Distal posterior process with 1–2 apical small setae and one bigger subapical seta; proximal posterior process bare and angular. Posterior margin proximally from medial bristle with 3-4 bigger gradually diminishing bristles followed by 2–3 smaller setae; internal surface with a stronger seta next to the medial bristle. Basal angle almost right-angled; basal margin with few setae. Ejaculatory apodeme with campanulate base and without rim. Aedeagus mostly triangular-shaped, apically widened. Aedeagal guides: 1) with two lamellae, 2) with wide and shallow lateral impressions, and 3) apically rounded, not extending beyond aedeagus distally. Aedeagal apodemes laterally angular.

#### Intraspecific variation.

The wide and compressed posterior impression of the gonocoxite, the distinct warts on the posterior process of the ventral branch of gonostylus and almost equal posterior processes of the dorsal branch of the gonostylus allow the species to be safely distinguished. In Finnish material, spine 2 on the ventral branch of the gonostylus is more massive than described by Laštovka (1972). In some specimens the base of the ejaculatory apodeme and the lateral margin of the dorsal branch of the gonostylus resemble those of *Mycetophila ichneumonea*. Phylogenetic analysis based on molecular data revealed three different clades (Fig. 4) that cannot be distinguished based on morphology (see also Discussion).

#### Hosts and distribution.

*Mycetophila uninotata* has been reared from species of *Collybia*, *Cortinarius* and *Lactarius* (Yakovlev 1994). Widely distributed in Central and Northern Europe but seems to be absent in the Mediterranean (Chandler 2013). The distribution gap between Central and Eastern Europe (e.g. absence in Poland, Belarus, Ukraine) can be explained by insufficient collecting.

## Discussion

This study represents the first evaluation of morphology-based species delimitation of fungus gnats by applying DNA sequence data. Results of the analyses, based on molecular data obtained for seven out of the nine European species from the *Mycetophila ruficollis* group, mostly supported the morphological species delimitation outlined by Laštovka (1972) and Laštovka and Kidd (1975). The genetic distance between members of different species, calculated from the COI barcoding region exceeded 2.9%, with intraspecific distance remaining below 1%. Our results are thus in accordance with inter- and intraspecific variation in different insect orders, documented to be on average over 2% and under 1%, respectively (e.g. Hebert et al. 2004, 2010). Only in *Mycetophila uninotata* the intraspecific distance was 2.3%, whereas three strongly supported clades were distinguished within this species in the phylogenetic trees. This evidence suggests that the current circumscription of *Mycetophila uninotata* includes two to three cryptic species. However, we were not able to find any morphological differences distinguishing these clades, each of which includes geographically distant material. More samples are needed to evaluate the genetic heterogeneity within *Mycetophila uninotata*.

The barcoding region of COI provided a clear barcoding gap for the distinction of all species, except for those in the described subclade of *Mycetophila ichneumonea* and *Mycetophila uninotata* that seems to include several recently differentiated species. In general, both the inter- and intraspecific variation remained lower than observed in other groups of insects (Angélica et al. 2014, Wang et al. 2012, Schwarzfeld and Sperling 2014). The comparison of the three regions sequenced from two genes revealed that the variation in the barcoding region of COI was slightly higher than in the following stretch of comparable length. The latter has been widely used in taxonomic studies on fungus gnats, focusing on higher taxonomic levels (Rindal et al. 2009, Ševčík et al. 2013). Here we showed its applicability also for species discrimination. By contrast, ITS2 rDNA that has become widely used in delimitation of insect taxa (e.g. Rokas et al. 2002, Schwarzfeld and Sperling 2014, Haarto and Ståhls 2014), included much less variation than each of the COI regions. Analyses of ITS2 rDNA data also resulted in lower phylogenetic resolution with some of the species remaining unresolved. Outperformance of COI over ITS2 has been observed also in Ichneumonidae (Schwarzfeld and Sperling 2014) and Mycetophilidae (Kurina et al. 2015).

Identification of gnats in the *Mycetophila ruficollis* group on the basis of morphological characters is complicated due to considerable intraspecific, yet only limited interspecific variation, mostly observed only upon examination of male genitalia. It was found to be most difficult to distinguish *Mycetophila strobli* and *Mycetophila uninotata* from *Mycetophila ichneumonea*. Also Laštovka (1972) mentioned that *Mycetophila uninotata* is most similar to *Mycetophila ichneumonea*. The phylogenetic analyses revealed close relationship of *Mycetophila uninotata* and *Mycetophila ichneumonea*, with lowest distance between the COI barcodes of their members. The difficulties in identification are obviously caused by high variation within the current concept of *Mycetophila uninotata*, suggested here to comprise cryptic species. COI data revealed *Mycetophila strobli* as more distant, but it appeared indistinguishable from *Mycetophila ichneumonea* based on ITS2 sequences. The morphologically distinct members of geographically separated populations, observed in some species, were not distinguished in molecular analyses. For example, some specimens of *Mycetophila idonea* from Georgia and Poland differed morphologically from their conspecifics but appeared homogeneous in the sequence data. Regarding geographic ranges, five out of the nine European species (viz. *Mycetophila evanida*, *Mycetophila ichneumonea*, *Mycetophila strobli*, *Mycetophila ruficollis* and *Mycetophila uninotata*) are widely distributed in the region while the rest of them (viz. *Mycetophila britannica*, *Mycetophila idonea*, *Mycetophila sepulta* and *Mycetophila suffusala*) have more scattered or endemic distribution patterns. Our study adds five new country records (viz. *Mycetophila evanida* from Estonia, *Mycetophila strobli* from Norway, and *Mycetophila ichneumonea*, *Mycetophila idonea* and *Mycetophila ruficollis* from Georgia) which widen the known distribution ranges, yet not changing the known patterns.

Our results suggest that several of the species in the *Mycetophila ruficollis* group have distinct host ranges. Thus far the larval stages of all European species in the group, except for *Mycetophila suffusala*, had been reported to feed from fruit bodies of a variety of mushrooms (Hackman and Meinander 1979, Yakovlev 1994, Chandler and Ribeiro 1995, Chandler 2010, Ševčík 2010), without preference for any fungal taxa. However, our data support the tendency apparent from literature records suggesting that gnats in the *Mycetophila ruficollis* group do not or only occasionally consume fruit bodies of the Boletales. Our results do not agree with the suggestion by Laštovka (1972) that polytypic *Mycetophila ichneumonea* consists of two to three subspecies. However, taken the difficulties in distinguishing this species from the *Mycetophila uninotata* complex, as reported here, it is possible that previous authors have had a mixture of species under consideration.

Combining morphological and molecular characters for identification of fungus gnats reared from identified fungal fruit bodies provides unique information of host use (Põldmaa et al. 2015). Most of the fungus gnats from the *Mycetophila ruficollis* group reared during recent years in Estonia from more than 680 fruit bodies represented *Mycetophila ichneumonea*. While not of frequent occurrence, *Mycetophila ichneumonea* was reared mostly from fruit bodies of two phylogenetically distant genera: *Lactarius* and *Cortinarius*. Such ‘disjunct host range’ is typical for several phytophagous insects (e.g. Bernays and Chapman 1994, Janz and Nylin 1997) but has so far not been described for mycetophages. Molecular data confirmed the conspecificity of adults reared from these two host genera with no host-related variation observed in the ITS2 or COI sequences. Other species, obtained by rearing of adults, were generally feeding on hosts from other fungal genera. While larvae of *Mycetophila ruficollis* consumed mostly saprotrophic mushrooms, *Mycetophila idonea* was reared from a single fruit body of *Amanita*. *Mycetophila strobli* seemed to prefer species from the earliest diverged lineages in the genus *Russula*, recognised as the subgenus *Compactae* by most authors. Taken the extensive rearing experiments from diverse mushroom taxa collected from the pine-dominated boreal forests we consider the observed host use patterns to represent fungus gnats’ specialisation in this habitat. Apparently, different fungi can serve as (preferred) hosts of members of the *Mycetophila ruficollis* group in other forest types.

Identification of closely related fungus gnats, as reported here in the *Mycetophila ruficollis* group, relies to large extent on a few morphological characters, mostly those of male terminalia. Blurred by intraspecific variation and the lack of such features in females, unambiguous identification is often impossible. Molecular data overcomes these obstacles and should be considered in species delimitation of fungus gnats. For that purpose, we advocate the use of the barcoding region of COI. Special value should be given to sequencing adults reared from identified fungi as these enable to elucidate host as well as geographic range of individual species of fungus gnats.

## Supplementary Material

XML Treatment for Mycetophila britannica

XML Treatment for Mycetophila evanida

XML Treatment for Mycetophila ichneumonea

XML Treatment for Mycetophila idonea

XML Treatment for Mycetophila ruficollis

XML Treatment for Mycetophila sepulta

XML Treatment for Mycetophila strobli

XML Treatment for Mycetophila suffusala

XML Treatment for Mycetophila uninotata

## References

[B1] Angélica M Gutiérrez C Vivero RJ Vélez ID Porter CH Uribe S (2014) DNA Barcoding for the Identification of Sand Fly Species (Diptera, Psychodidae, Phlebotominae) in Colombia. PLoS ONE 9(1): . doi: 10.1371/journal.pone.0085496, PMCID: PMC3893204 10.1371/journal.pone.0085496 PMC3893204 24454877

[B2] Bechev D (2000) World distribution of the genera of fungus gnats (Diptera: Sciaroidea, excluding Sciaridae). Studia Dipterologica 7: 543–552.

[B3] Beebe NW Saul A (1995) Discrimination of all members of the *Anopheles punctulatus* complex by polymerase chain reaction-restriction fragment length polymorphism analysis. American Journal of Tropical Medicine and Hygiene 53: 478–481. 7485705 10.4269/ajtmh.1995.53.478

[B4] Bernays EA Chapman RF (1994) Host-plant Selection by Phytophagous Insects. Chapman and Hall, New York, 312 pp. doi: 10.1007/b102508

[B5] Chandler PJ Ribeiro E (1995) The Sciaroidea (Diptera) of the Atlantic Islands. Boletim do Museu Municipal do Funchal (História Natural), Suplemento No. 3: 1–170.

[B6] Chandler PJ Bechev DN Caspers N (2005) The Fungus Gnats (Diptera: Bolitophilidae, Diadocidiidae, Ditomyiidae, Keroplatidae and Mycetophilidae) of Greece, its islands and Cyprus. Studia Dipterologica 12: 255–314.

[B7] Chandler P (2010) Associations with Fungi, Mycetozoa and Plants. In: Chandler P (Ed.) A Dipterist’s Handbook. The Amateur Entomologist, Vol. 15 Orpington, Kent, 417–441.

[B8] Chandler PJ (2013) Fauna Europaea: Mycetophilidae. In: Beuk P Pape T (Eds) Fauna Europaea: Diptera, Nematocera. Fauna Europaea, version 2.6. http://www.faunaeur.org [accessed 10.iii.2015]

[B9] Folmer O Black M Hoeh W Lutz R Vrijenhoek R (1994) DNA primers for amplification of mitochondrial cytochrome C oxidase subunit I from diverse metazoan invertebrates. Molecular Marine Biology and Biotechnology 3: 294–299. 7881515

[B10] Haarto A Ståhls G (2014) When mtDNA COI is misleading: congruent signal of ITS2 molecular marker and morphology for North European *Melanostoma* Schiner, 1860 (Diptera, Syrphidae). ZooKeys 431: 93–134. doi: 10.3897/zookeys.431.7207 25152670 10.3897/zookeys.431.7207 PMC4141176

[B11] Hackman W Meinander M (1979) Diptera feeding as larvae on macrofungi in Finland. Annales Zoologici Fennici 16: 50–83.

[B12] Hajibabaei M Smith MA Janzen DH Rodriguez JJ Whitefield JB Hebert PDN (2006) A minimalist barcode can identify a specimen whose DNA is degraded. Molecular Ecology Notes 6: 959–964. doi: 10.1111/j.1471-8286.2006.01470.x

[B13] Hebert PD Cywinska A Ball SL deWaard JR (2003) Biological identifications through DNA barcodes. Proceedings of the Royal Society B: Biological Sciences 270: 313–321. doi: 10.1098/rspb.2002.2218 10.1098/rspb.2002.2218 PMC1691236 12614582

[B14] Hebert PDN Stoeckle MY Zemlak TS Francis CM (2004) Identification of birds through DNA barcodes. PLoS Biology 2(10): . doi: 10.1371/journal.pbio.0020312 10.1371/journal.pbio.0020312 PMC518999 15455034

[B15] Hebert PDN deWaard JR Landry JF (2010) DNA barcodes for 1/1000 of the animal kingdom. Biology letters 6: 359–362. doi: 10.1098/rsbl.2009.0848 20015856 10.1098/rsbl.2009.0848 PMC2880045

[B16] Hippa H Kurina O (2012) New species and new records of Afrotropical *Manota* Williston (Diptera, Mycetophilidae), with a key to the species. Zootaxa 3455: 1–48.

[B17] Jakovlev J (2014) Checklist of the fungus gnats of Finland: Bolitophilidae, Diadocidiidae, Ditomyiidae, Keroplatidae and Mycetophilidae (Diptera). ZooKeys 441: 119–149. doi: 10.3897/zookeys.441.7646 25337013 10.3897/zookeys.441.7646 PMC4200453

[B18] Janz N Nylin S (1997) The role of female search behaviour in determining host plant range in plant feeding insects: a test of the information processing hypothesis. Proceeding of the Royal Society of London B: Biological Sciences 264: 701–707. doi: 10.1098/rspb.1997.0100

[B19] Katoh K Toh H (2008) Recent developments in the MAFFT multiple sequence alignment program. Brief Bioinform 9(4): 286–98. doi: 10.1093/bib/bbn013 18372315 10.1093/bib/bbn013

[B20] Kjærandsen J (2012) Checklist of Nordic fungus gnats (Diptera: Bolitophilidae, Diadocidiidae, Ditomyiidae, Keroplatidae, Mycetophilidae and *Sciarosoma*). 1.0. http://sciaroidea.info/node/48341 [Updated 14.02.2014]

[B21] Kurina O (1991) Mycetophilidae (Diptera) reared from macrofungi in Estonia. Proceedings of the Estonian Academy of Sciences. Biology 40: 89–90.

[B22] Kurina O (1994) New records of Mycetophilidae (Diptera) reared from macrofungi in Estonia. Proceedings of the Estonian Academy of Science. Biology 43: 216–220.

[B23] Kurina O (1996) Hibernation of fungus gnats (Diptera, Mycetophilidae) in Estonian caves. Studia Dipterologica 3: 221–229.

[B24] Kurina O (2003) Notes on the Palaearctic species of the genus *Polylepta* Winnertz (Diptera: Mycetophilidae) with a new synonymization. Entomologica Fennica 14: 91–97.

[B25] Kurina O Õunap E Ramel G (2011) *Baeopterogyna mihalyii* Matile (Diptera, Mycetophilidae): association of sexes using morphological and molecular approaches with the first description of females. ZooKeys 114: 15–27. doi: 10.3897/zookeys.114.1364 21976994 10.3897/zookeys.114.1364 PMC3130343

[B26] Kurina O Oliveira SS (2013) The first *Cordyla* Meigen species (Diptera, Mycetophilidae) from continental Australia and Tasmania. ZooKeys 342: 29–43. doi: 10.3897/zookeys.342.6045 24194654 10.3897/zookeys.342.6045 PMC3817428

[B27] Kurina O Õunap E Põldmaa K (2015) Two new *Neuratelia* Rondani (Diptera: Mycetophilidae) species from Western Palaearctic: a case of limited congruence between morphology and DNA sequence data. ZooKeys 496: 105–129. doi: 10.3897/zookeys.496.9315 25931957 10.3897/zookeys.496.9315 PMC4410159

[B28] Krivosheina NP Zaitzev AI Jakovlev JB (1986) Insects as decomposers of macrofungi in the forests of the European part of USSR. Nauka, Moscow, 309 pp [In Russian]

[B29] Laffoon J (1957) A revision of the Nearctic species of *Fungivora* (Diptera, Mycetophilidae). Iowa State College Journal of Science 31: 141–340.

[B30] Lane J (1955) Neotropical *Mycetophila* Meigen excluding those of the Chilean Subregion (Diptera, Mycetophilidae). Transactions of the Royal Entomological Society of London 106: 393–420. doi: 10.1111/j.1365-2311.1955.tb00782.x

[B31] Laštovka P (1963) Beitrag zur Kenntnis der Europäischen *Fungivora*-Arten aus der Gruppe *vittipes* (Zett.) (Diptera, Fungivoridae). Časopis Ceskoslovenské Společnosti Entomologické 60: 312–327.

[B32] Laštovka P (1972) Holarctic species of *Mycetophila ruficollis*-group (Diptera, Mycetophilidae). Acta Entomologica Bohemoslovaca 69: 275–294.

[B33] Laštovka P Kidd L (1975) Review of the British and notes on other species of the *Mycetophila ruficollis*-group with the description of a new species (Dipt., Mycetophilidae). Entomologist’s Monthly Magazine 110: 203–214.

[B34] Martinsson S Kjærandsen J Sundberg P (2011) Towards a molecular phylogeny of the fungus gnat genus *Boletina* (Diptera: Mycetophilidae). Zoologica Scripta 40: 272–281. doi: 10.1111/j.1463-6409.2011.00474.x

[B35] Matile L (1980) Superfamily Mycetophiloidea 15. Family Mycetophilidae. In: Crosskey RW (Ed.) Catalogue of the Diptera of the Afrotropical Region. British Museum (Natural History), London, 216–230.

[B36] Merz B Haenni JP (2000) Morphology and terminology of adult Diptera (other than terminalia). In: Papp L Darvas B (Eds) Contributions to a Manual of Palaearctic Diptera. Volume 1 Science Herald, Budapest, 21–51.

[B37] Oliveira SS Amorim DS (2014) Catalogue of Neotropical Diptera. Mycetophilidae. Neotropical Diptera 25: 1–87. doi: 10.1007/s13744-013-0171-z

[B38] Õunap E Viidalepp J (2009) Description of *Crypsiphona tasmanica* sp. nov. (Lepidoptera: Geometridae: Geometrinae), with notes on limitations in using DNA barcodes for delimiting species. Australian Journal of Entomology 48: 113–124. doi: 10.1111/j.1440-6055.2009.00695.x

[B39] Põldmaa K Jürgenstein S Bahram M Teder T Kurina O (2015) Host diversity and trophic status as determinants of species richness and community composition of fungus gnats. Basic and Applied Ecology 16: 46–53. doi: 10.1016/j.baae.2014.10.004

[B40] Ribeiro E (1990) Contribution to the study of fungus-gnats (Diptera: Mycetophiloidea) of Portugal. II – seven new records. Boletim da Sociedade Portuguesa de Entomologia 118: 173–194.

[B41] Rindal E Søli GEE Bachmann L (2009) On the systematics of the fungus gnat subfamily Mycetophilinae (Diptera): a combined morphological and molecular approach. Journal of Zoological Systematics and Evolutionary Research 47: 227–233. doi: 10.1111/j.1439-0469.2008.00498.x

[B42] Rokas A Nylander JAA Ronquist F Stone GN (2002) A maximum-likelihood analysis of eight phylogenetic markers in gallwasps (Hymenoptera: Cynipidae): implications for insect phylogenetic studies. Molecular Phylogenetics and Evolution 22: 206–219. doi: 10.1006/mpev.2001.1032 11820842 10.1006/mpev.2001.1032

[B43] Schwarzfeld MD Sperling FAH (2014) Species delimitation using morphology, morphometrics, and molecules: definition of the *Ophion scutellaris* Thomson species group, with descriptions of six new species (Hymenoptera, Ichneumonidae). ZooKeys 462: 59–114. doi: 10.3897/zookeys.462.8229 25589855 10.3897/zookeys.462.8229 PMC4284433

[B44] Ševčík J (2010) Czech and Slovak Diptera associated with fungi. Slezské zemské muzeum, Opava, 112 pp.

[B45] Ševčík J Kaspřák D Tóthová A (2013) Molecular phylogeny of fungus gnats (Diptera: Mycetophilidae) revisited: position of Manotinae, Metanepsiini, and other enigmatic taxa as inferred from multigene analysis. Systematic Entomology 38: 654–660. doi: 10.1111/syen.12023

[B46] Ševčík J Kaspřák D Mantič M Ševčíková T Tóthová A (2014) Molecular phylogeny of the fungus gnat family Diadocidiidae and its position within the infraorder Bibionomorpha (Diptera). Zoologica Scripta 43(4): 370–378. doi: 10.1111/zsc.12059

[B47] Simon C Frati F Beckenbach A Crespi B Liu H Flook P (1994) Evolution, weighting, and phylogenetic utility of mitochondrial gene sequences and a compilation of conserved polymerase chain reaction primers. Annals of the Entomological Society of America 87(6): 651–701. doi: 10.1093/aesa/87.6.651

[B48] Søli GEE Vockeroth JR Matile L (2000) A. 4. Families of Sciaroidea. In: Papp L Darvas B (Eds) Contributions to a Manual of Palaearctic Diptera. Appendix. Science Herald, Budapest, 49–92.

[B49] Swofford DL (2003) PAUP*. Phylogenetic Analysis Using Parsimony (* and other methods), Version 4. Sinauer Associates, Sunderland, Massachusetts.

[B50] Tamura K Stecher G Peterson D Filipski A Kumar S (2013) MEGA6: Molecular Evolutionary Genetics Analysis Version 6.0. Molecular Biology and Evolution 30(12): 2725–2729. doi: 10.1093/molbev/mst197 24132122 10.1093/molbev/mst197 PMC3840312

[B51] Wang G Li C Guo X Xing D Dong Y Wang Z Zhang Y Liu M Zheng Z Zhang H Zhu X Wu Z Zhao T (2012) Identifying the Main Mosquito Species in China Based on DNA Barcoding. PLoS ONE 7(10): . doi: 10.1371/journal.pone.0047051 10.1371/journal.pone.0047051 PMC3468562 23071708

[B52] Waugh J (2007) DNA barcoding in animal species: progress, potential and pitfalls. BioEssays 29: 188–197. doi: 10.1002/bies.20529 17226815 10.1002/bies.20529

[B53] Wu H (1997) A study on the *Mycetophila ruficollis* group (Diptera: Mycetophilidae) from China. Entomotaxonomia 192: 117–129.

[B54] Yakovlev EB (1994) Palearctic Diptera associated with fungi and mycomycetes. Karelian Research Center, Russian Academy of Sciences, Forest Research Institute, Petrozavodsk, 127 pp [In Russian with English summary]

[B55] Zaitzev AI (1999) New species of the *Mycetophila vittipes*-group from Russia (Diptera: Mycetophilidae). Zoosystematica Rossica 8: 183–187.

[B56] Zaitzev AI (2003) Fungus gnats (Diptera, Sciaroidea) of the fauna of Russia and adjacent regions. Part II. An international Journal of Dipterological Research 14: 77–386.

